# Morphology and distribution of the external labial sensilla in Fulgoromorpha (Insecta: Hemiptera)

**DOI:** 10.1007/s00435-012-0174-z

**Published:** 2012-09-30

**Authors:** Jolanta Brożek, Thierry Bourgoin

**Affiliations:** 1 Department of Zoology, University of Silesia, Bankowa 9, 40-007 Katowice, Poland; 2 Département Systématique and Evolution, Museum National d’Histoire Naturelle, UMR 7205, MNHN-CNRS, 45 rue Buffon, 75005 Paris, France

**Keywords:** Fulgoromorpha, Apex labium, Labial sensilla distribution, Phylogeny, Gustatory function, Exaptation

## Abstract

The present paper describes the sensory structures on the apical segment of the labium in fifteen fulgoromorphan families (Hemiptera: Fulgoromorpha), using the scanning electron microscope. Thirteen morphologically distinct types of sensilla are identified: five types of multiporous sensilla, four types of uniporous sensilla and four types of nonporous sensilla. Three subapical sensory organ types are also recognized, formed from one to several sensilla, each characteristic of a family group. Sensilla chaetica (mechanoreceptive sensilla) fall into three categories dependent on length and are numerous and evenly distributed on the surface of the labium except where they occur on specialized sensory fields. The planthopper morphological ground plan is represented by two apical pair of sensory fields (dorsal and ventral) on which 11 dorsal pairs of sensilla (10 peg-like pairs + 1 specialized pair dome or cupola-like) and 2 ventral pairs of sensilla basiconica occur. Two main patterns (cixiid and issid) together with more specialized ones (derbid, lophopid, flatid and fulgorid) are reported. Disparity and diversity of the sensory structures are analyzed from a taxonomic and functional perspective. A gustatory function is provided for several chemoreceptive labial sensilla, as in the antennal flagellum sensilla in some other Hemiptera. This represents a more recently evolved function for the planthopper labium. Finally, further lines of study are suggested for future work on the phylogeny of the group based on the studied characters.

## Introduction

Insect sensilla consist of an exocuticular outer structure by or through which stimuli are conveyed to one or more sensory cell processes within the sensilla. Shape and size of the cuticular outer structures vary, but most sensilla appear as hairs or pegs. In such sensilla, the socket region can be distinguished from the shaft of the hair or peg (Altner and Prillinger 1980). When sensilla have been examined on the insect body, antennal and mouthpart sensilla have attracted the most attention.

In insects, the antennae represent the primarily sensory structures and are usually richly endowed with sensilla. Most of these are generally distributed on the flagellum and exhibit a variety of forms and characteristics in relation to their various functions of contact chemoreception, mechanoreception and thermohygroreception (Chapman 1998). With regards Fulgoromorpha, most studies on antennal sensilla have focused on putative olfactory sensilla, located on the pedicel (reviewed in Bourgoin and Deiss 1994; Stroiński et al. 2011). Indeed, planthopper flagellum sensilla are less in number than in many other insect, and it is of interest that typical chemoreceptors seem to be absent. This absence is probably compensated for by the olfactory sensilla of the pedicel. In this respect, the low number of sensilla on the flagellum should be regarded as a possible functional specialization of the flagellum itself (Romani et al. 2009).

In Hemiptera, the mouthparts have evolved into a rostrum consisting of a short, conical and dorsal labrum and a longer segmented labium, bisected by a stylet groove within which lie the outer mandibular and inner maxillary stylets. The labial tip is bilobed with each right and left lobe exhibiting symmetrically distributed sensilla grouped into sensory fields (Foster et al. 1983b; Backus 1985). There are no chemoreceptors on the stylets that enter the tissue of the host. The only sensilla associated with the food canal are those of the cibarium (Foster et al. 1983a) and precibarium (Backus and McLean 1982, 1983, 1985). Consequently, the insects do not taste the food they are about to feed on until they begin to ingest it. In fact, the majority of the chemoreceptors found on the labium in Hemiptera are in a position enabling them only to monitor chemicals from the external surface of the host (Miles 1958; Backus 1988; Chapman 1995). For these primarily phytophagous insects, these labial sensilla provide the only direct link with the host-plant (Cobben 1988), and it is supposed that the receptors found in the labial tip provide the insects with information that influences their subsequent feeding behavior (Foster et al. 1983b). Labial sensilla might therefore selectively reflect evolution of these host-plant patterns and relationships (Attié et al. 2008) and simultaneously reflect the phylogenetical evolution of the group; accordingly, their study should bring some taxonomic/phylogenetic signal that has not yet been tested with respect to these issues.

The labial sensilla of most hemipterans can apparently perform both chemosensory and mechanosensory functions, while dabbing with the labium, during plant surface exploration. According to Backus (1988), Heteroptera and Auchenorrhyncha are rather similar in their labial sensory system while these two groups show a greater diversity and variability than in Sternorrhyncha while Cobben (1988) indicated more diversity than expected, in Fulgoromorpha. In the latter group, the structure, function and classification of labial sensilla have been precisely studied in only two delphacid crop pests, *Nilaparvata lugens* by Foster et al. (1983a, b) and *Peregrinus maidis* by Backus (1985), and found to be similar. A very brief description of the labial sensillae was also published for another delphacid, *Tagosodes orizicolus* (Mora et al. 2001). In addition, a special subapical sensory organ was first reported by Sogawa (1977) and formally recognized by Cobben (1988) in various species in delphacids, dictyopharids and tettigometrids but probably absent in fulgorids. The purpose of this study is to investigate these and new characters referred to here as the labial sensory apparatus with the objectives of (1) completing a systematic description of the sensory structures and provide a terminology for future work and (2) to evaluate the usefulness of these characters for identifying taxa and for phylogenet studies in Fulgoromorpha.

## Materials and methods

The study was based on dry material from the collections of the Museum National d’Historie Naturelle in Paris (MNHN). SEM photographs were taken from various species representing 15 families of Fulgoromorpha. The list of examined species is provided in Table 3. In the species marked with an asterisk (*), only the sub-apical sensilla were analyzed.

The specimens were coated in gold–palladium and photographed with a Jeol JSM 840 A.

The tips of the chemoreceptor dendrites can be covered by a viscous fluid (containing mucopolisacharides), which sometimes exudes through the terminal pore or wall pores of the sensillum (Chapman 1998). This was observed in the SEM photographs as artefacts visible on or around the peg gustatory, olfactory or contact-chemoreceptive sensilla.

### Terminology

Sensilla of insects occur in two classes as mechanosensitive or chemosensitive sensilla. Their terminology varies according to their function and morphology including ultrastructure, systems and features that do not overlap. The external morphology is based on the criteria established by Altner and Prillinger (1980), Zacharuk (1980) and Foster et al. (1983b). The different types of sensilla are given with their abbreviations (manly from Brożek and Chłond 2010) in Tables 1 and 2.

**Table 1:** Terminology and definition of sensilla used in the present paper

Category	Function	Pore	Sensilla type
Contact-chemoreceptive sensilla (bimodal sensilla)	Gustatory and tactile	UP: uniporous with one (sub-) apical pore	Sensilla basiconica: bristle- (BRSN1, BRSN2) or cone-like sensilla (BSN1, BNS2) with a flexible basal socket; 3–10 internal sensory neurons (Chapman 2003)
Chemoreceptive sensilla	Gustatory	UP: uniporous with one terminal pore	Peg-like (PGSU1, PGSU2, PPSU) or clavate-like sensilla (CLSU), in an inflexible socket; 3–10 internal sensory neurons (Chapman 2003)
Chemoreceptive sensilla	Olfactive and/or thermic	MP: multiporous	Variously shaped sensilla (OPSM, CUSM, DSSM), peg-like (PGSM, PGSMC), tubular (ECLT), branched or multilobed (TEBM), placoid (PFPL). Over the surface but sunken into an inflexible socket
Mechanoreceptive sensilla	Tactile	NP: no pore (or one basal molting pore)	Sensilla chaetica: sharp tip haired sensilla in a basal flexible socket of various length (CH1, CH2, CH3); often with cuticular sculturing

*BSN1* sensillum basiconicum, nonporous, long, *BSN2* sensillum basiconicum, nonporous, short, *BRSN1* bristle-like sensillum, nonporous, long, *BRSN2* bristle-like sensillum, nonporous, short, *OPSM* oval plate sensillum, multiporous, *PGSM* peg sensillum, multiporous, *PGSMC* peg sensilla, multiporous, complex, *CUSM* cupola-shaped sensillum, multiporous, *DSSM* dome-shaped sensillum, multiporous, *PPSU* peg-in-pit sensillum, uniporous (sensillum coeloconicum), *PGSU1* peg sensillum, uniporous, long, *PGSU2* peg sensillum, uniporous, short, *CLSU* clavate sensillum, uniporous, *Sa-ECLT*, elevated, cone-like to tubular sensillum, *Sa-TEBM* tubular, branched, multilobated sensillum; *Sa-PFPL* placoid flattened, *Sa-PMPL* placoid, multilobated, sensillum with numerous minute lobes, *CH1* sensillum chaeticum, long, *CH2* sensillum chaeticum, medium, *CH3*, sensillum chaeticum, short, *p* pore, *ml* molting pore, *Soc.Fl* flexible sockets, *Gr* grooved surface of sensillum chaeticum, *Lg* labial groove, *SF-D* dorsal sensory field, *SF-V* ventral sensory field, *SF-D-A* dorsal sensory field A, *SF-D-B* dorsal sensory field B, *Mx* maxillae, *Md* mandibulae, *RS* right side of the labial tip, *LS* Left side of the labial tip

**Table 2:** Type, number and length of sensilla chaetica and sensilla of the labial tip in fulgoromorphan families

	Labial apical segment: mechanosensitive sensilla			Apical sensilla: mechanoreceptive or/and contact-chemoreceptive sensilla				Apical sensilla: chemosensitive sensilla, gustatory				Apical sensilla: chemosensitive sensilla, olfactory				Number of the apical sensilla
Types of the sensilla	CH1	CH2	CH3	BSN1	BSN2	BRSN1	BRSN2	PPSU	PGSU1	PGSU2	CLSU	CUSM	DSSM	PGSM	PGSMC OPSM	
Length of the sensilla	45–250 μm	20–45 μm	1.0–10 μm	10–20 μm	1.0–10 μm	30–40 μm	10–30 μm	1.5 μm	5.0–7.0 μm	2.0–5.0 μm	2.0 μm	1.5 μm	0.82 μm	2.5 μm	10.8 μm PGSMC	
Delphacidae	—	+	+	—	2	—	—	1	1	8	—	—	1	—	—	13
Cixiidae	—	+	+	—	2	—	—	1	—	9	—	—	1	—	—	13
Achilidae	—	+	—	1	1	—	—	1	2	6	—	—	1	—	—	12
Meenoplidae	—	—	+	—	1	—	—	—	—	9	—	1	—	—	—	12
Kinnaridae	—	+	—	1	1	—	—	1	—	9	—	—	1	—	—	13
Derbidae	+	+	+	—	1	—	—	1	—	4 + 6	—	—	—	?	—	12
Ricaniidae	+	+	—	7	2	—	—	—	3	6	—	—	—	1	—	19
Issidae	+	+	—	2	4	—	—	—	—	—	8	—	—	—	1 PGSMC	15
Flatidae	+	+	+	12	3	—	—	—	—	16	—	—	—	1	—	32
Tropiduchidae	—	+	—	1	4	—	—	—	—	9	—	—	—	1	—	15
Lophopidae	+	+	—	2	30	—	—	—	—	3	—	—	—	?	—	35
Nogodinidae	+	+	—	4	2	—	—	—	—	8	—	—	—	1	1 OPSM	16
Dictyopharidae	+	+	—	1	2	—	—	—	9	—	—	—	—	1	—	13
Fulgoridae	+	+	—	—	—	10	15	—	3	6	—	—	—	1	—	35
Tettigometridae	—	+	—	—	2	—	—	—	12	—	—	—	—	?	—	14

*Symbol key*: ?, no multiporous sensilla found; +, sensilla present; —, sensilla absent

## Results

### Distribution of the sensilla on the apical segment of the labium

In planthoppers, the labial sensory apparatus is formed by a set of subapical and apical sensilla that show a wide diversity of cuticular structures and organization. They are distributed in various sensory fields and in different locations. Their topography allows us to recognize the following two main groups.

#### Apical sensilla on the tip of the labium

In most cases, these sensilla are located in sensory fields (SF) that are clearly differentiated from the rest of the labium tip, forming isolated convex or concave areas. The following two types are recognized: a dorsal sensory field (SFD) situated on each side of the apex of the labial groove and one medial or two paired ventral sensory fields (SFV). Sensilla are usually more numerous on the dorsal rather than ventral fields. Thirteen different morphological types of sensilla have been found on the labial tip (BSN1, BNS2, BRSN1, BRSN2, PGSM, PGSMC, OPSM, CUSM, DSSM, PPSU, PGSU1, PGSU2, CLSU).

#### Sub-apical sensilla on the labium

On each side near the tip of the labium, one or more sensilla are present. These correspond to the ‘preapical sensory organ’ described by Cobben (1988). They are situated into a more or less deep cavity. Three morphologically distinct sensilla types have been recognized (Sa-ECLT, Sa-TEBM, Sa-PFPL). They are multiporous sensilla, usually surrounded by numerous sensilla chaetica (CH1, CH2, CH3).

### Morphological characters of the sensilla of the apical segment of the labium

#### Mechanoreceptive NP sensilla or contact-chemoreceptive UP sensilla: tactile and gustatory sensilla at the tip of the labium

**Fig. 1:**
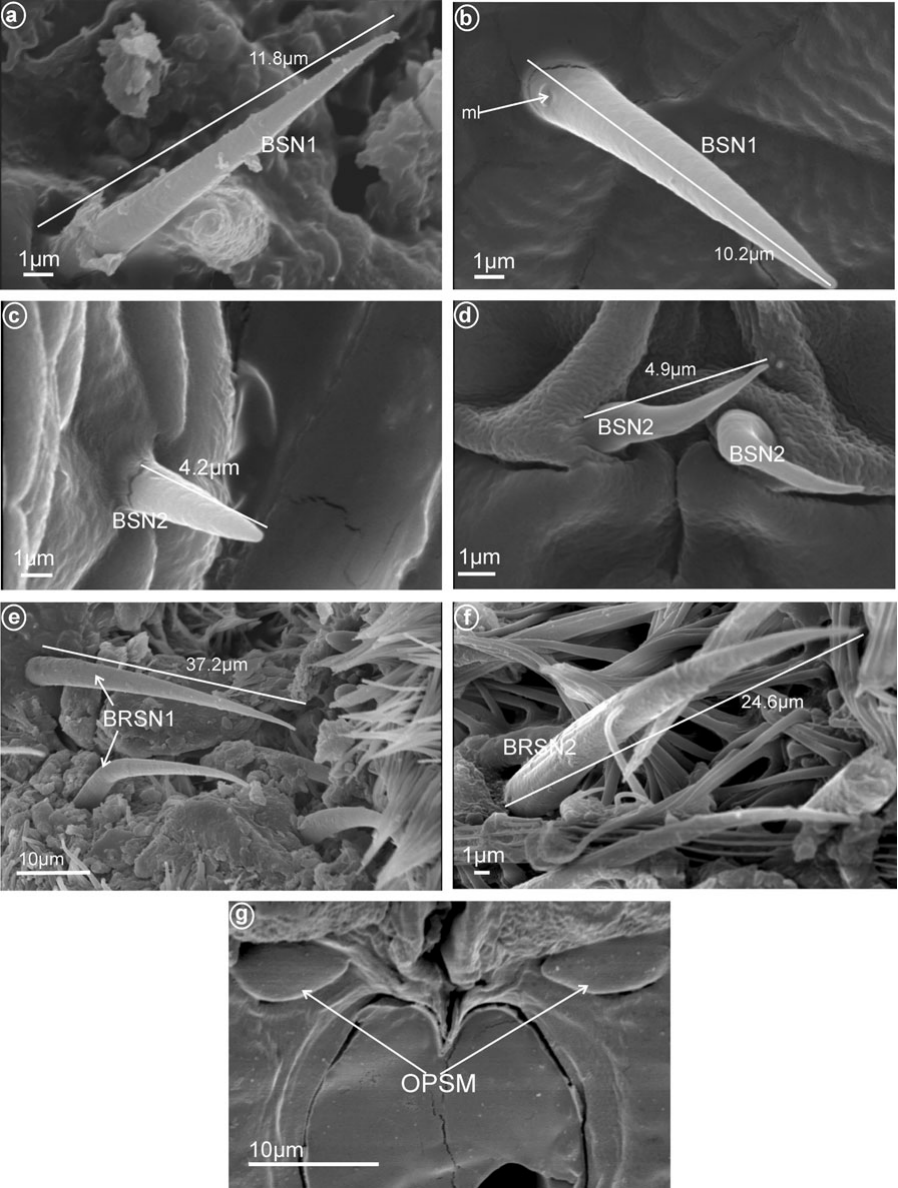
Types and size of the apical sensilla of Fulgoromorpha. **a, b** BSN1 sensillum basiconicum. **c, d** BSN2 sensilla basiconica. **e** BRSN1 bristle-like sensilla. **f** BRSN2 bristle-like sensillum. **g** OPSM oval plate sensilla

These sensilla are distinctly inserted within sockets and their bases are flexible. They are wider basally and gradually tapper to apex with the tip slightly rounded. The cuticular walls are smooth with a molting pore near the base (Fig. 1a–d). No terminal pore is visible, and therefore, these sensilla should be considered as mechanoreceptive sensilla. However, their characteristics and location suggest they may be contact-chemoreceptive sensilla with an indistinct terminal pore. While sensilla basiconica (BSN1, BSN2) are present in most fulgoromorpha families examined, bristle-like sensilla (BRSN1, BRSN2) are found only in Fulgoridae. These non-porous(?) sensilla are long, tapered toward apex, end in a fine-spun tip and have a molting pore at their base. The cuticular wall shows evident grooves. Sensilla basiconica and bristle-like sensilla are respectively subdivided into two groups according to their size as follows:

- *Large* (*10–20* μm) *sensilla basiconica, nonporous* (BSN1, Fig. 1a, b). These sensilla were identified in Kinnaridae (Fig. 9e, sensillum no. 13, *L* = 9.4 μm), Ricaniidae (Fig. 11g, h, sensilla no. 11–16, *L* = 15.7 μm, *L* = 18.4 μm), Issidae (Fig. 12b, sensilla no. 14, 15, *L* = 11.5 μm), Flatidae (Fig. 13e, f, *L* = 14.0 μm, *L* = 16.2 μm), Tropiduchidae (Fig. 14c, d, h, sensillum no. 14, *L* = 0.6 μm), Lophopidae (Fig. 15b, d, h, sensillum no. 34, 35, *L* = 9.7 μm), Nogodinidae (Fig. 16f–h, sensilla no. 10, 11, 14, 15, *L* = 11.1 μm, *L* = 9.7 μm) and Dictyopharidae (Fig. 17d, h, sensillum no. 12, *L* = 14 μm).

**Fig. 2:**
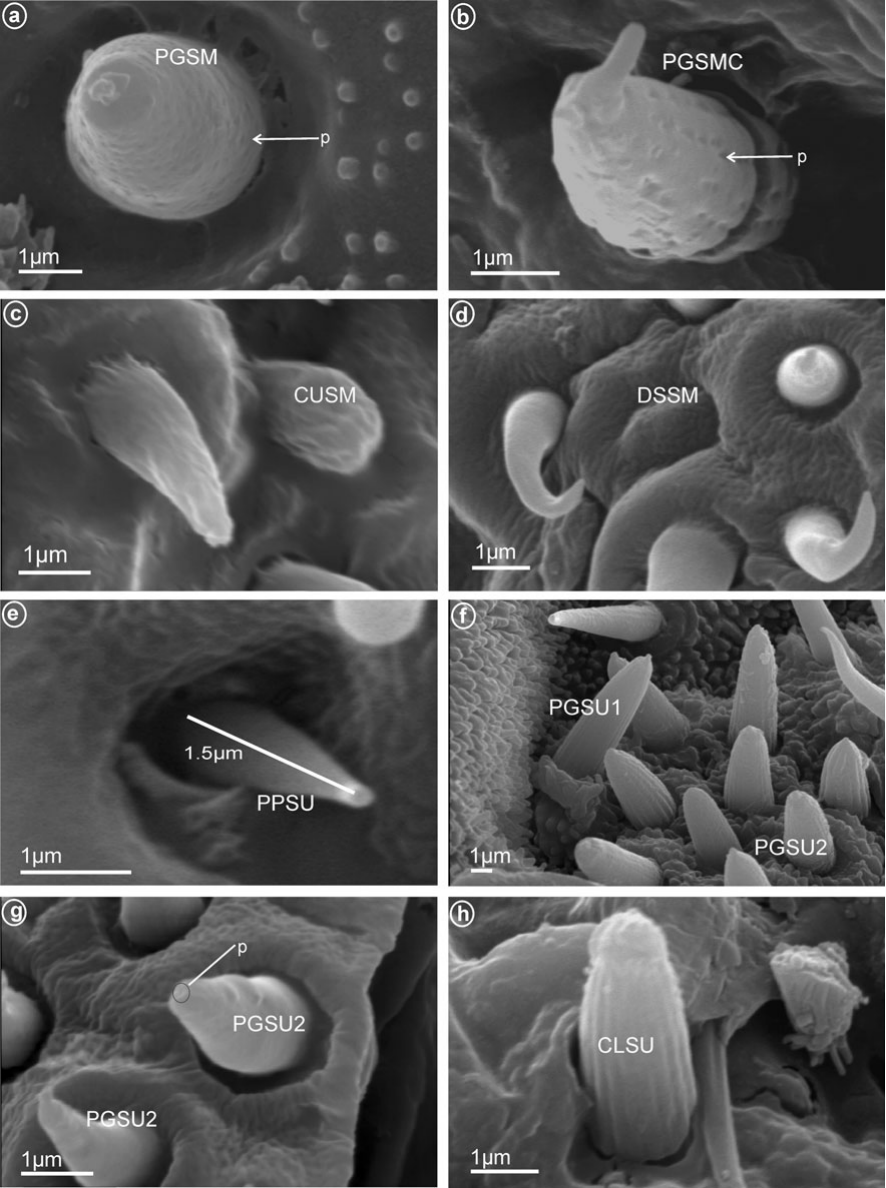
Types of the apical chemosensilla of Fulgoromorpha. **a** PGSM peg sensillum. **b** PGSMC peg sensillum, complex. **c** CUSM cupola-shaped sensillum. **d** DSSM dome-shaped sensillum. **e** PPSU peg-in-pit sensillum. **f** PGSU1 peg sensillum. **g** PGSU2 peg sensillum. **h** CLSU clavate sensillum, p pore

**Fig. 3:**
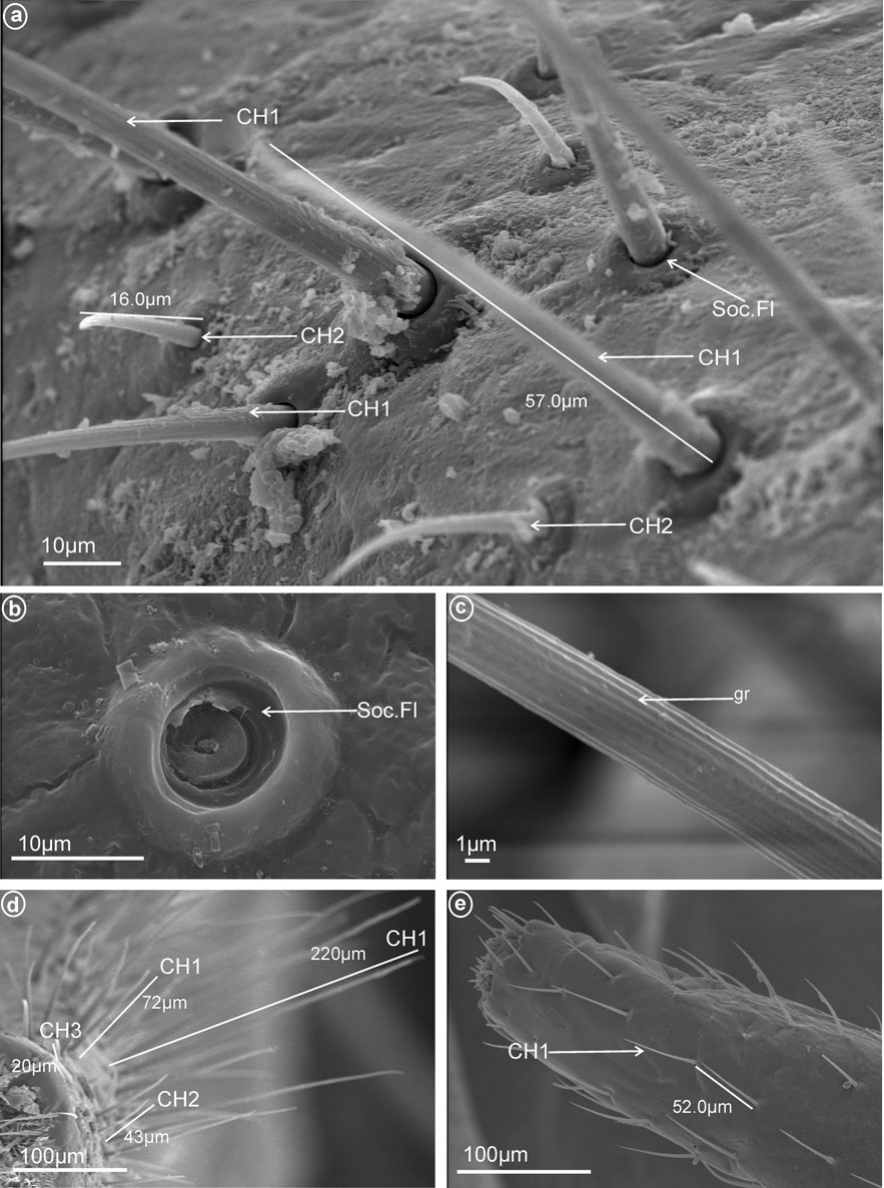
Types of the mechanosensilla of fulgoromorphan families. **a** CH1 sensillum chaeticum, CH2 sensillum chaeticum. **b** Soc.Fl flexible sockets. **c** Gr grooved surface of sensillum chaeticum. **d** CH3 sensillum chaeticum, sensilla CH1, CH2, CH3 densely arranged. **e** Sensillum chaeticum CH1 widely separated

**Fig. 4:**
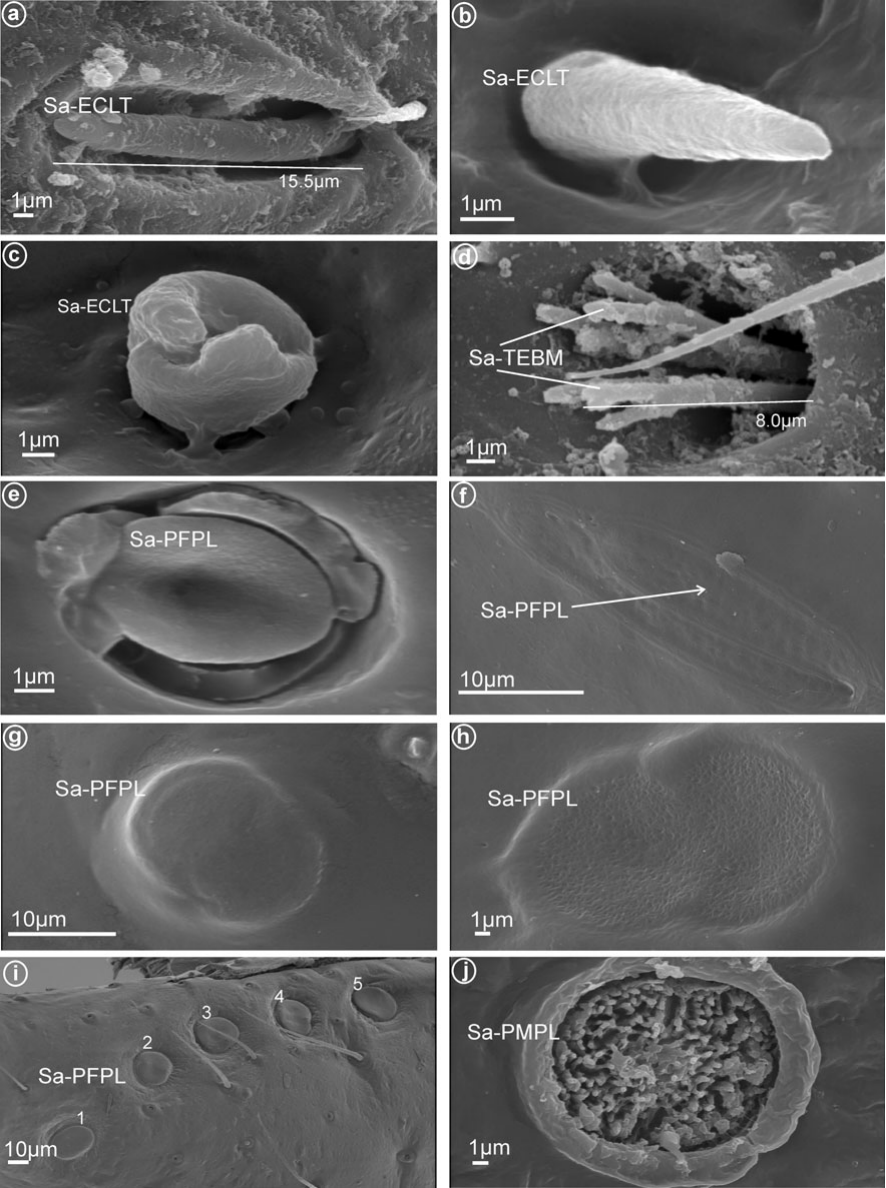
Shapes of the subapical, multiporous labial sensilla in the fulgoromorphan families. **a** Sa-ECLT elevated, cone-like to tubular sensillum (Cixiidae). **b** Sa-ECLT elevated, cone-like to tubular sensillum (Meenoplidae). **c** Sa-ECLT elevated, cone-like to tubular sensillum (Lophopidae). **d** Sa-TEBM tubular, branched, multilobated sensillum (Delphacidae). **e** Sa-PFPL placoid flattened sensillum (Achilidae). **f** Sa-PFPL placoid flattened sensillum (Dictyopharidae). **g** Sa-PFPL placoid flattened sensillum (Tropiduchidae). **h** Sa-PFPL placoid flattened sensillum (Ricaniidae). **i** Sa-PFPL placoid flattened sensilla (no. 1–5) (Nogodinidae). **j** Sa-PMPL placoid, multilobated, sensillum with numerous minute lobes (Flatidae)
- *Short* (*1.0–10* μm) *sensilla basiconica, nonporous* (BSN2, Fig. 1c, d). These sensilla were identified in Delphacidae (Fig. 5b, h, sensilla no. 12, 13, *L* = 4.9 μm), Cixiidae (Fig. 6a, e, sensilla no. 12, 13, *L* = 2.1 μm), Achilidae (Fig. 7d, sensillum no. 11), Meenoplidae Fig. 8c, d, sensillum no. 11, *L* = 2.6 μm), Kinnaridae (Fig. 9e, sensillum no. 12, *L* = 2.09 μm), Derbidae (Fig. 10b, g, sensillum no. 12, *L* = 4.5 μm), Ricaniidae (Fig. 11d, sensilla no. 17–19, *L* = 2.9 μm, *L* = 3.1 μm), Issidae (Fig. 12b–d, sensilla no. 10–13, *L* = 5.1 μm, *L* = 7.2 μm), Flatidae (Fig. 13d, *L* = 1.1 μm), Tropiduchidae (Fig. 14c, d, g, sensilla no. 2, 3, 13, 15, *L* = 4.1 μm), Lophopidae (Fig. 15c, g, sensilla no. 1–30, *L* = 3.7 μm), Nogodinidae (Fig. 16g, h, sensilla no. 13, 14, *L* = 2.7 μm), Dictyopharidae (Fig. 17d, g, h, sensilla no. 11, 13, *L* = 5.8 μm) and Tettigometridae (Fig. 19a, c, sensilla no. 13, 14, *L* = 6.8 μm).

**Fig. 5:**
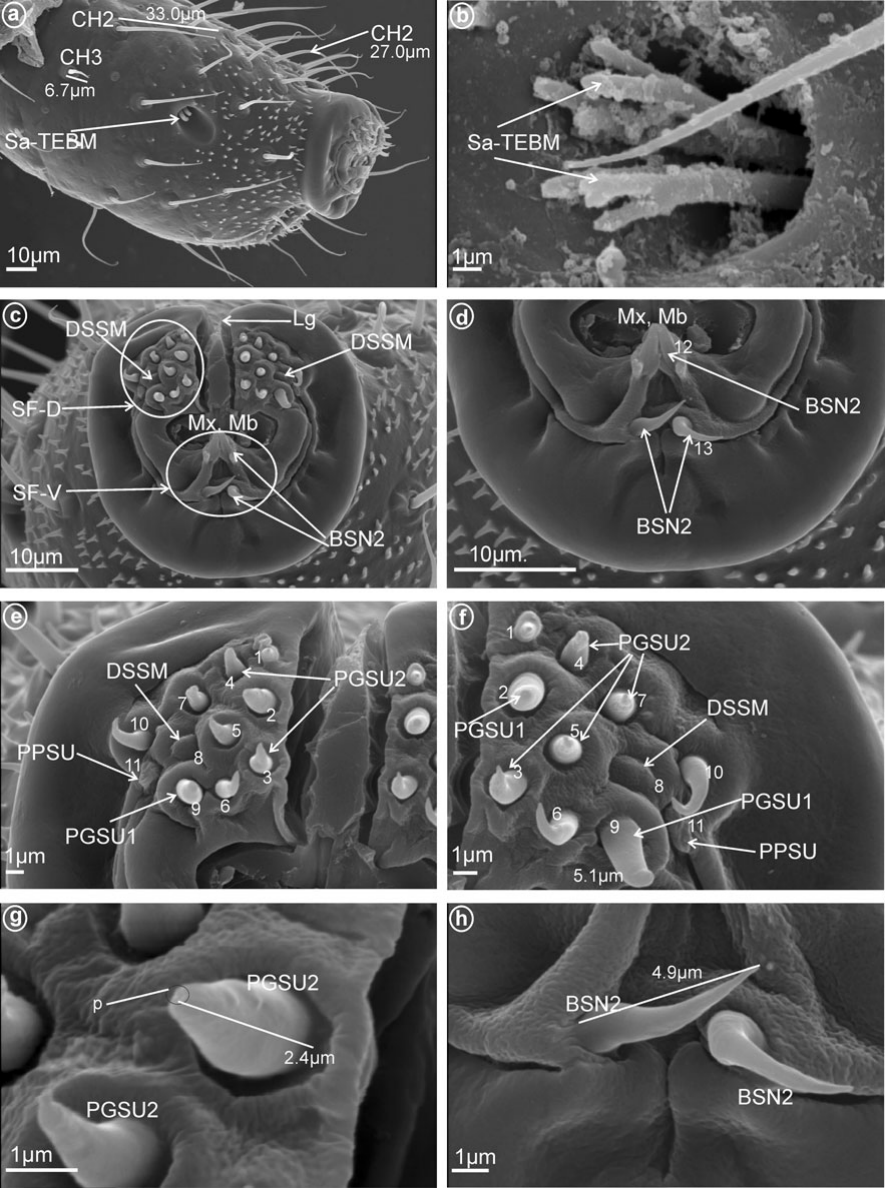
Types and arrangement of the labial sensilla of *Peregrinus maidis* (Delphacidae). **a** CH2, CH3, Sa-TEBM in lateral view. **b** Sa-TEBM (in enlargement). **c** SF-D with marked DSSM, SF-V with marked BSN2, Mx, Md. **d** Ventral sensory field with BSN2 (sensilla no. 12, 13). **e** *Right side* of the labial tip (SF-D): DSSM (sensillum no. 8), PPSU (sensillum no. 11), PGSU1 (sensilla no. 2, 9) and PGSU2 (sensilla no. 3–5, 7 and no. 1, 6, 10). **f** *Left side* of the labial tip, sensilla placed symmetrically with respect to the right. **g** Length of the PGSU2, p pore. **h** Length of the BSN2

**Fig. 6:**
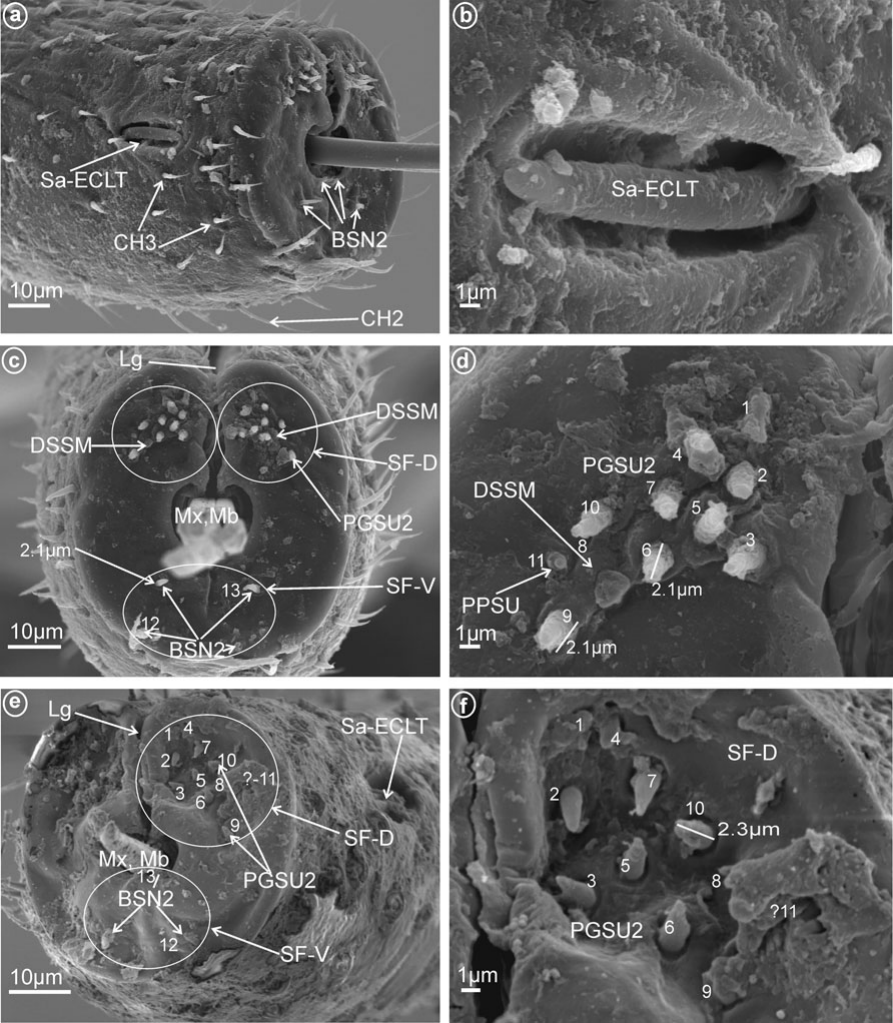
Types and arrangement of the labial sensilla of *Myndus taffini* **a**–**d** (Cixiidae: Cixiinae: Oecleini) and **e**–**f** *Brixidia boukokoensis* (Cixiidae: Brixidiinae: Brixiidini). **a** BSN2, CH2, CH3, Sa-ECLT in lateral view. **b** Sa-ECLT (in enlargement). **c** SF-D with visible DSSM and PGSU2, SF-V with visible BSN2 (sensilla no. 12, 13), Mx, Md, Lg. **d** PGSU2 (sensilla no. 1–**7**, 9, 10), PPSU (sensillum no. 11), DSSM (sensillum no. 8). **e** SF-D with PGSU2, PPSU (probably sensillum no. 11?), SF-V with visible BSN2 (sensilla no. 12, 13), Mx, Md, Lg. **f** Length of the PGSU2 (sensilla no. 1–**7**, 9, 10), PPSU (sensillum no. 11), DSSM (probably sensillum no. 8)

**Fig. 7:**
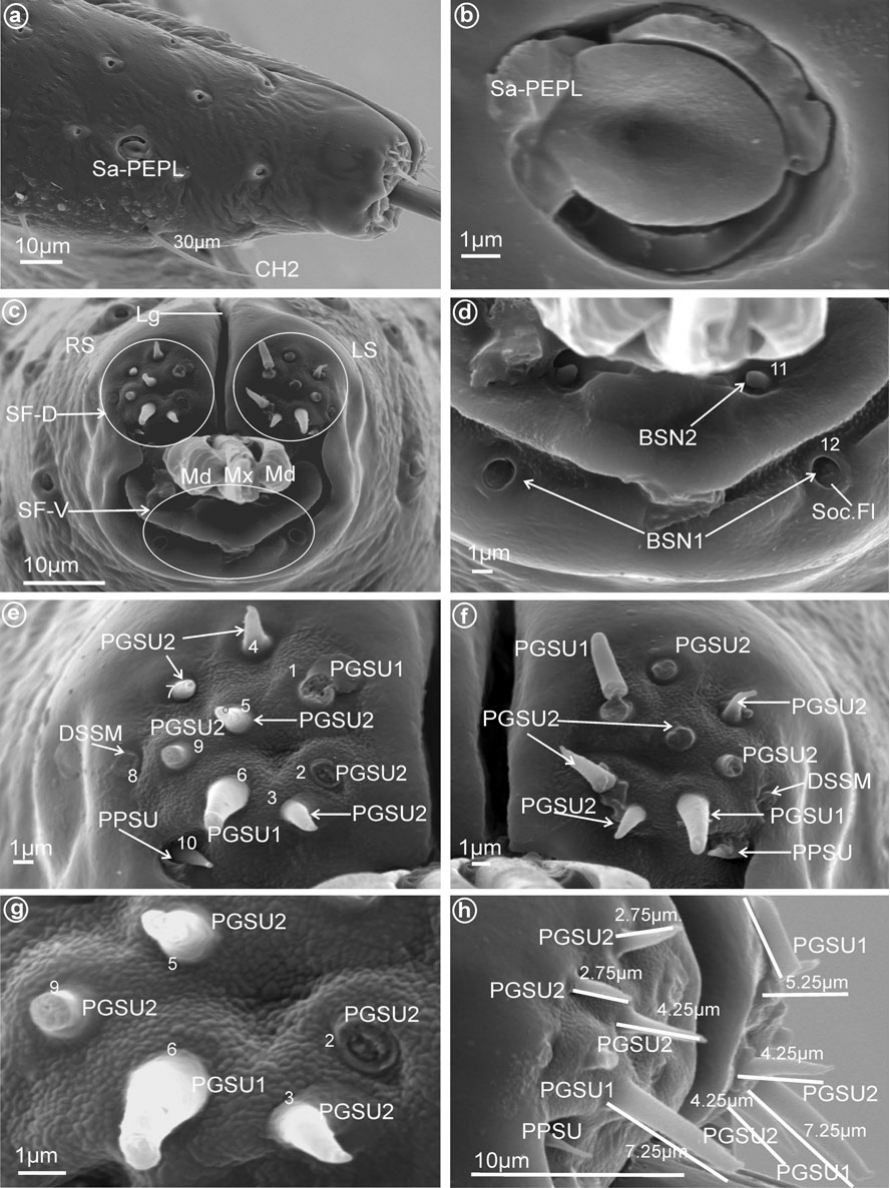
Types and arrangement of the labial sensilla of *Ballomarius kawandanus* (Achilidae). **a** CH2, Sa-PFPL in lateral view. **b** Sa-PFPL (in enlargement). **c** Lg, SF-D and SF-V, Mx, Md in front view. **d** Ventral sensory field with BSN1 (sensillum no. 12) and BSN2 (sensillum no. 11), Soc.Fl. **e** *Right side* of the labial tip (SF-D):PGSU1 (sensilla no. 1, 6), PGSU2 (sensilla no. 2–5, 7, 9), DSSM (sensillum no. 8) and PPSU (sensillum coeloconicum no. 10). **f** *Left side* of the labial tip, sensilla are arranged in the same way as for the right side. **g** PGSU2 and PGSU1 (in enlargement). **h** Length (μm) of the PGSU1 and PGSU2 sensilla

**Fig. 8:**
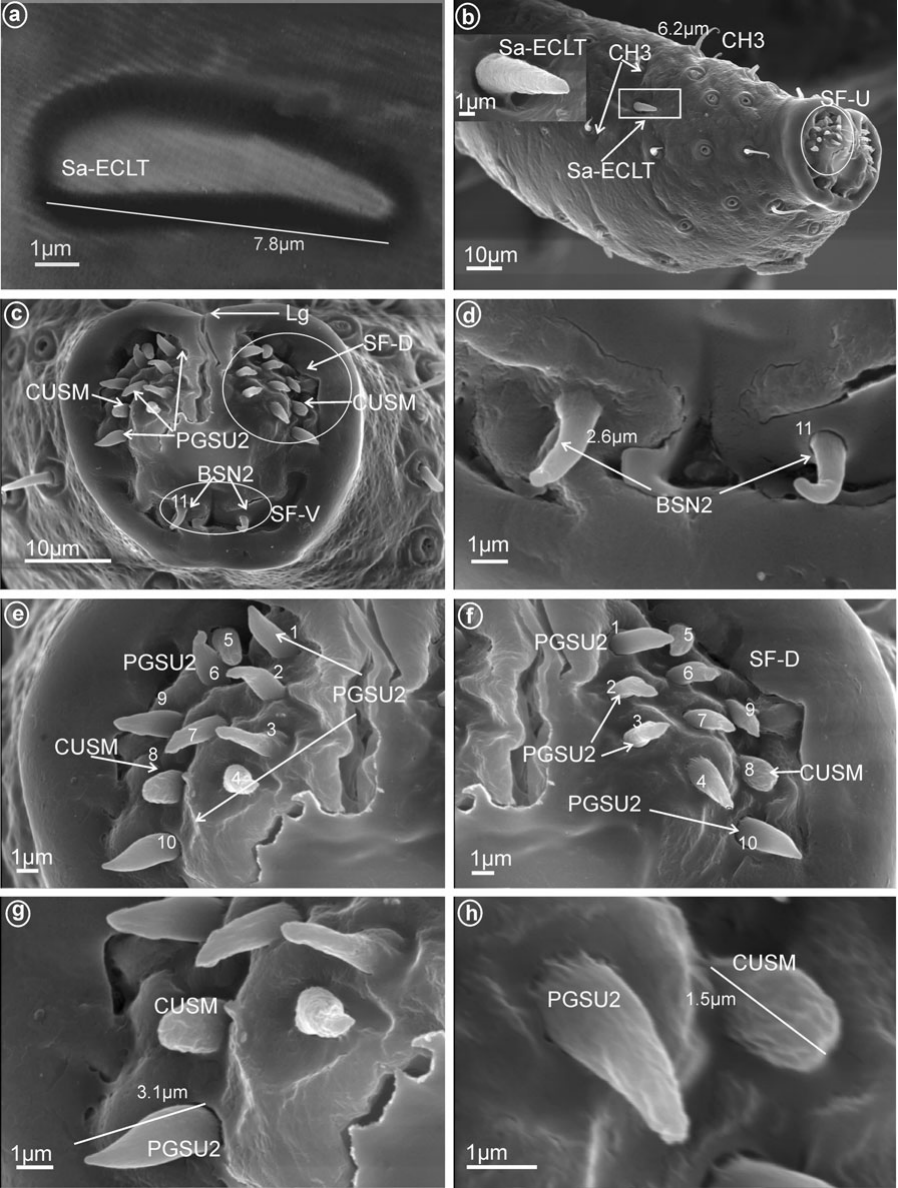
Types and arrangement of the labial sensilla of Meenoplidae. **a** *Kermesia immaculata*, length of the Sa-ECLT. **b**–**h** *Nisia nervosa*, **b** CH3, Sa-ECLT, sensilla in lateral view. **c** SF-D with PGSU2 and CUSM, SF-V with BSN2 (sensillum no. 11). **d** Ventral sensory field with BSN2 (length of the BSN2). **e, f** *Right* and *left* side of the labial tip, dorsal sensory fields with PGSU2 (sensilla no. 1–**7**, 9,10) and CUSM (sensillum no. 8). **g, h** Length (μm) of the PGSU2 and of the CUSM

**Fig. 9:**
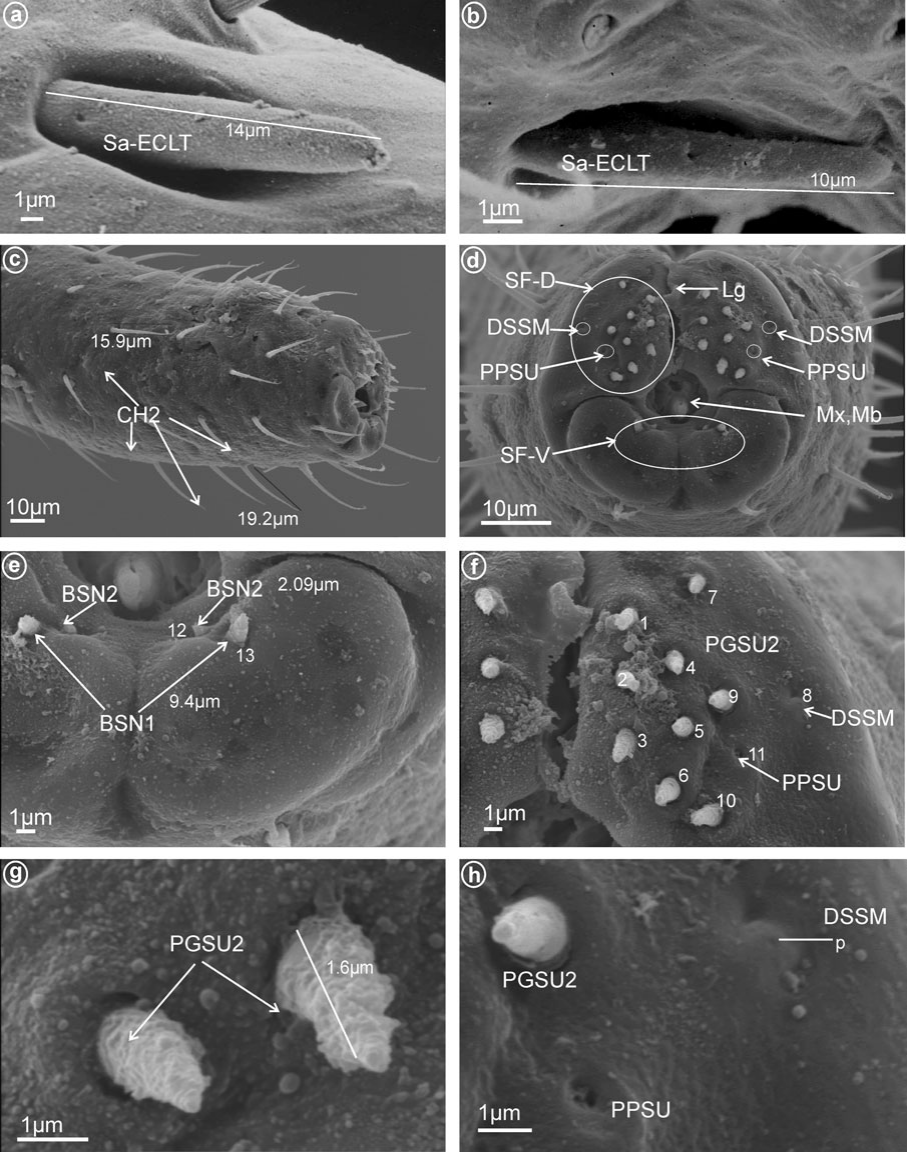
Types and arrangement of the labial sensilla of Kinnaridae. **a** *Southia capnorhina*, length of the Sa-ECLT. **b** *Atopocixius major,* length of the Sa-ECLT. **c-h** *Nesomircixia insularis*, **c** length of the CH2, sensilla spaced in series, evenly. **d** SF-D with visible DSSM and PPSU, SF-V is marked, Mx, Md. **e** Ventral sensory field with BSN1 (sensillum no. 13, length 9.4 μm) and BSN2 (sensillum no. 12, length 2.09 μm). **f** *Left side* of the labial tip: PGSU2 (sensilla no. 1–7, 9, 10), DSSM (sensillum no. 8) and PPSU (sensillum no. 11). **g** Length of the PGSU2. **h** Distribution of the DSSM and PPSU (sensillum coeloconicum), p-pores

**Fig. 10:**
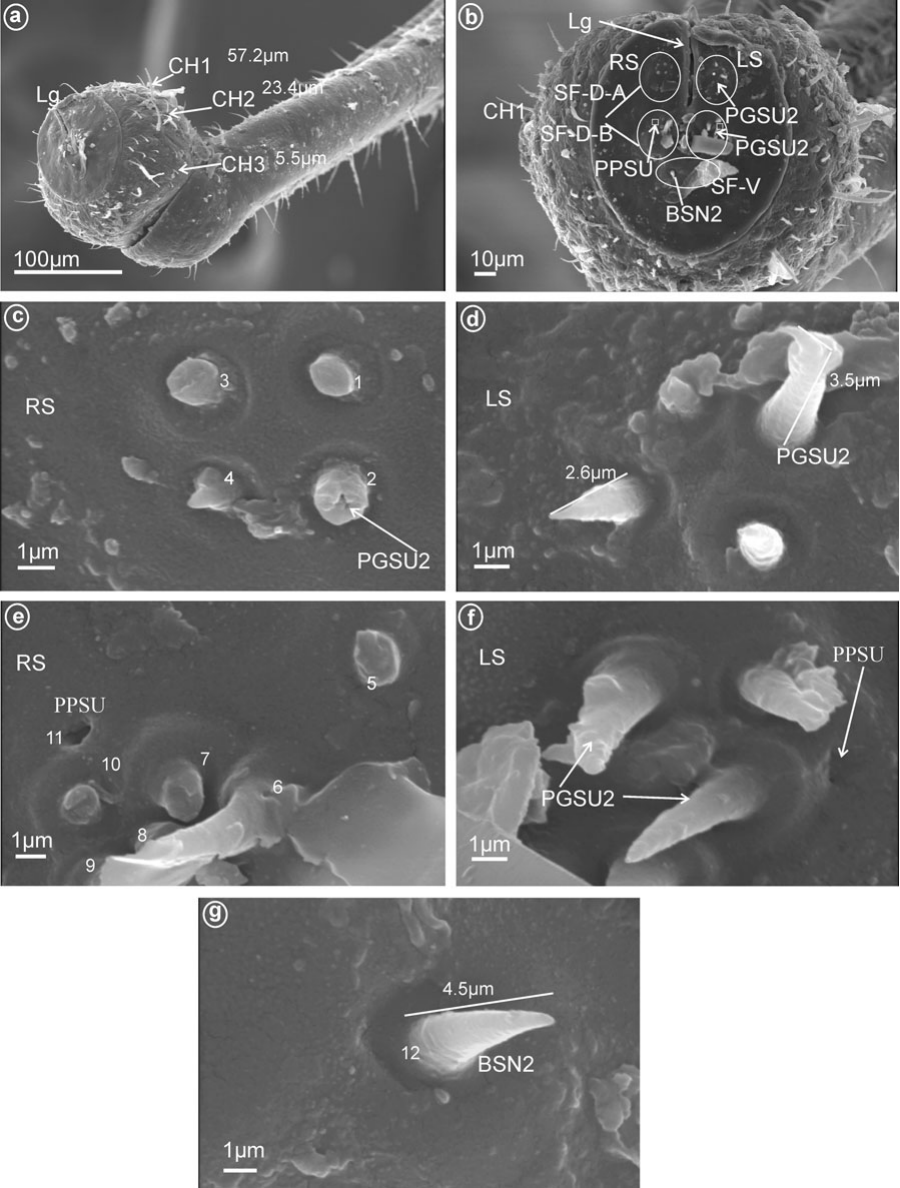
Types and arrangement of the labial sensilla of *Proutista fritillaris* (Derbidae). **a** Length of the CH1, CH2 and CH3. **b** SF-D-A (there are four PGSU2), SF-D-B (there are six PGSU2 and one PPSU), SF-V with BSN2. **c** *Right side* (RS) of the SF-D-A with PGSU2 (sensilla no. 1–4). **d** *Left side* (LS) of the SF-D-A, length of the PGSU2 sensilla. **(e)** RS, dorsal sensory field B, distribution of the PPSU (sensillum no. 11) and PGSU2 (sensilla no. 5–10). **f** PGSU2 and PPSU (in enlargement). **g** Length of the BSN2 (sensillum no. 12)

**Fig. 11:**
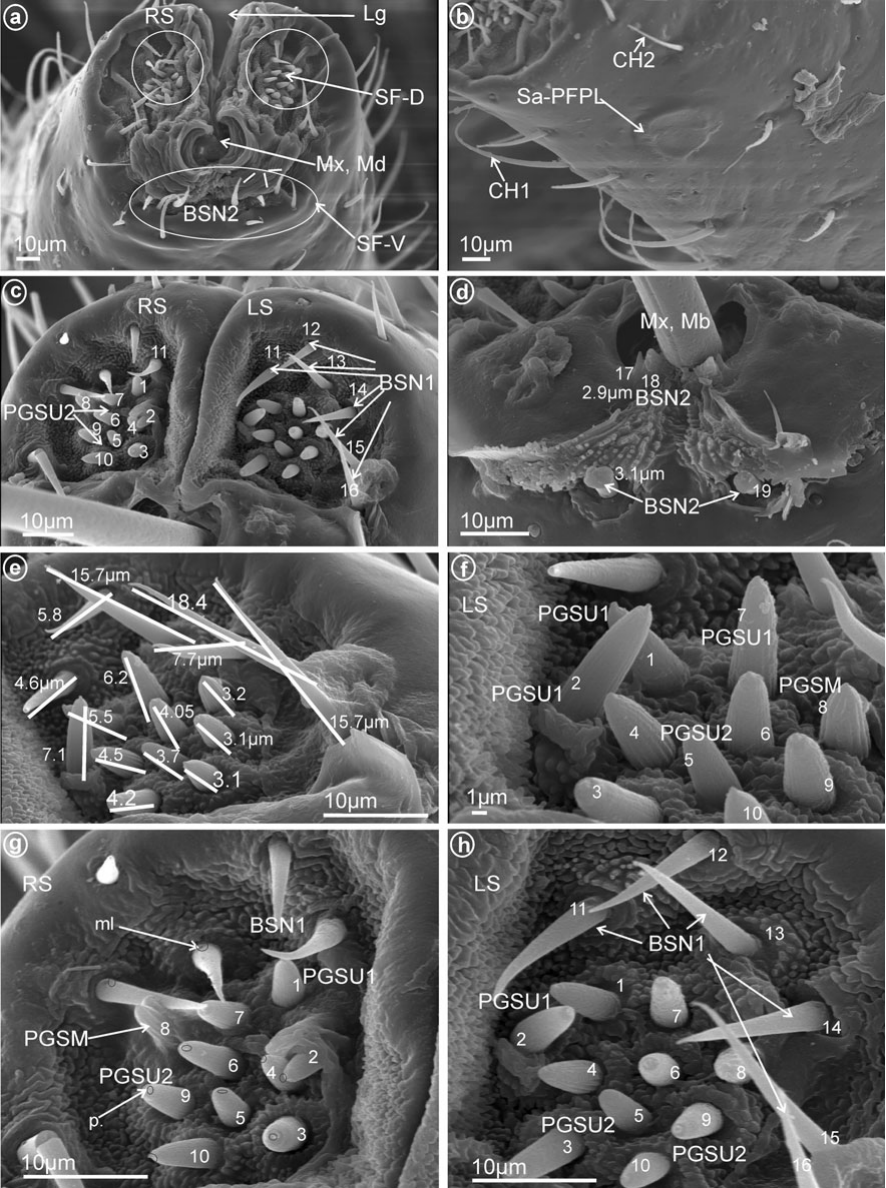
Types and arrangement of the labial sensilla of *Pochazia fuscata* (Ricaniidae). **a** Labial tip, in front view: Lg, SF-D, SF-V with BSN, Mx, Md, RS *right side* of the tip. **b** Distribution of the sensilla CH1, CH2 and Sa-PFPL. **c** Dorsal sensory fields: PGSU2 (sensilla no. 3–6, 9, 10) and BSN1 (sensilla no. 11–16). **d** Ventral sensory field with BSN2 (sensilla no. 17–19). **e** Length of individual sensilla. **f** Dorsal sensory field with PGSM (sensillum no. 8), PGSU1 (sensilla no. 1, 2, 7) and PGSU2 (sensilla no. 3–6, 9, 10). **g, h** Detailed view: BSN1, PGSU1, PGSU2 and PGSM on the *right* (RS) and the *left* (LS) side of the dorsal sensory fields, p pore, ml molting pore

**Fig. 12:**
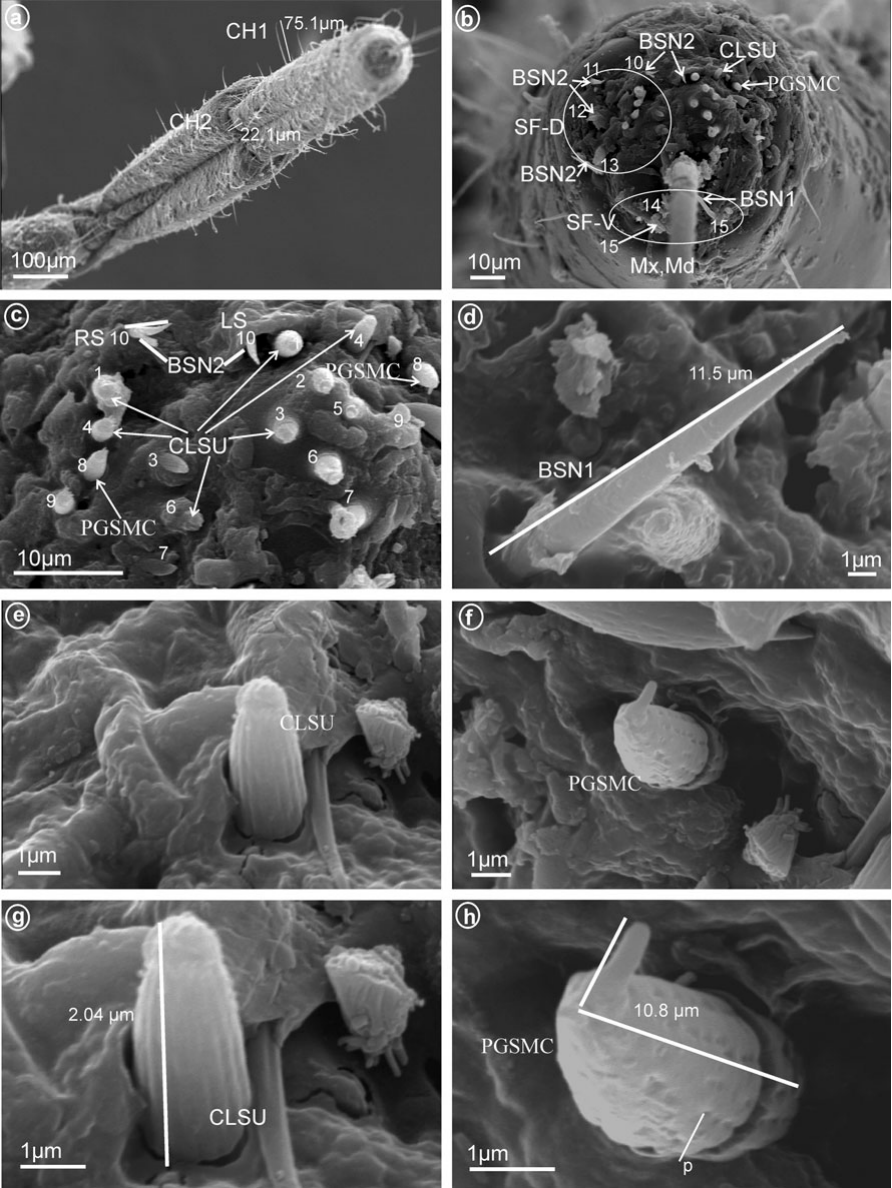
Types and arrangement of the labial sensilla of *Trienopa paradoxa* (Issidae). **a** Length of the CH1 and CH2. **b** Front view on: SF-D with sensilla BSN2 (no. 10–13), CLSU and PGSMC, SF-V with sensilla BSN1 (no. 14, 15), Mx, Md. **c** Right, dorsal sensory field with CLSU (sensilla no. 1–7, 9) and PGSMC (sensillum no. 8). **d** Length of the BSN1. **e** Shape of the CLSU. **f** Shape of the PGSMC. **g** Length of the CLSU. **h** Length of the PGSMC, p-pores are visible

**Fig. 13:**
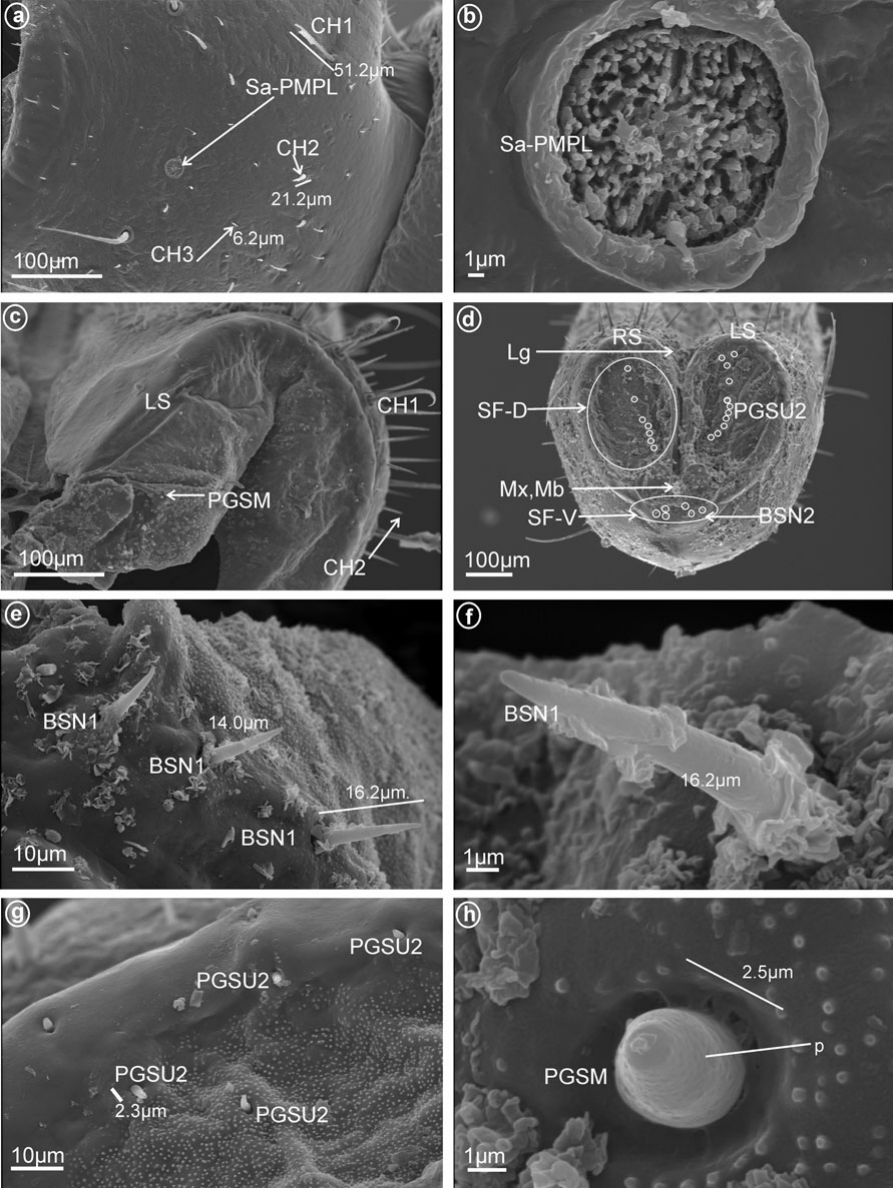
Types and arrangement of the labial sensilla of *Flatida* sp. (Flatidae). **a** Length of the CH2, CH3 and location of the Sa-PMPL. **b** Front view on the Sa-PMPL. **c** Dorsal sensory field with PGSM. **d** Front view on labial tip: *right* (RS) and *left* (LS) tip, Lg, PGSU2 are marked on dorsal sensory field (SF-D), SF-V with the BSN2, Mx, Md. **e** Length and distribution of the BSN1 in the dorsal sensory field. **f** Detailed view on surface of the BSN1. **g** Distribution and size of the PGSU2. **h** Detailed view on surface of the PGSM, p-pores are visible

**Fig. 14:**
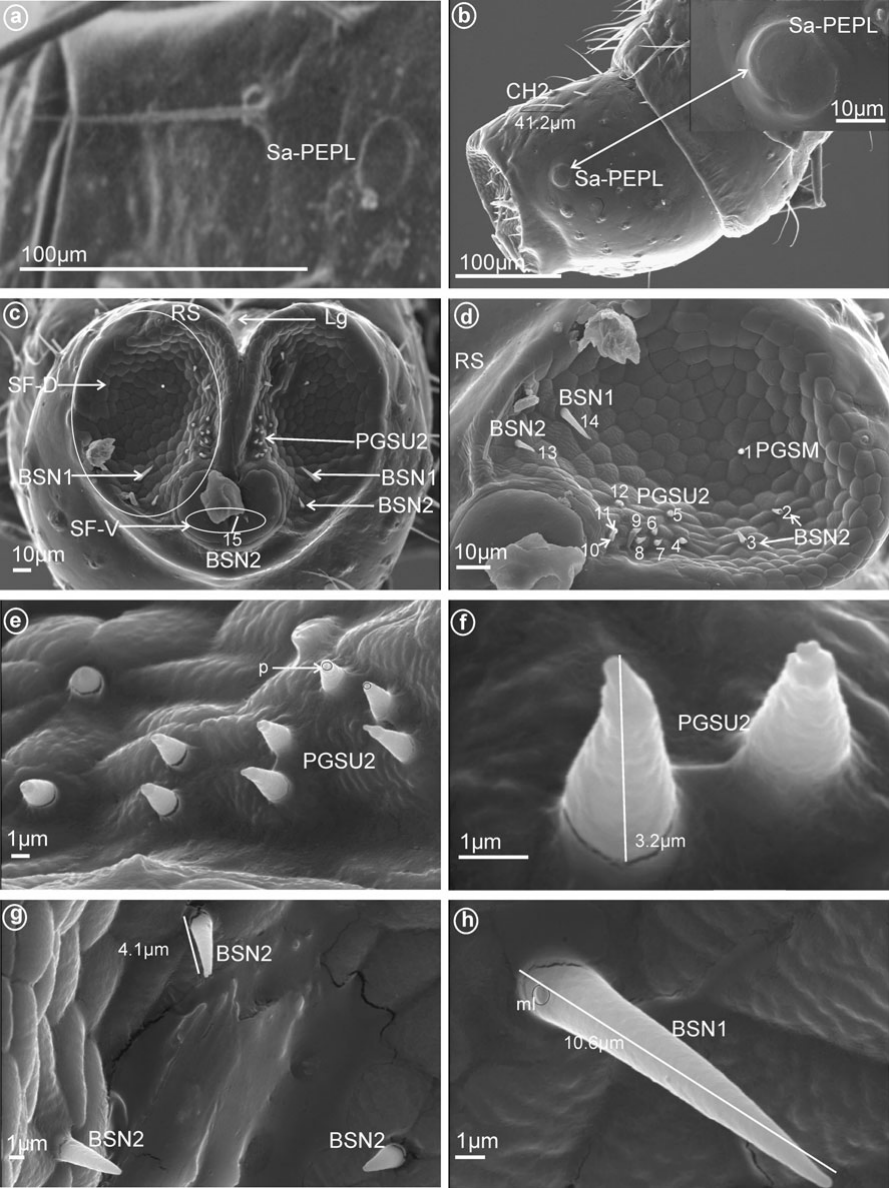
Types and arrangement of the labial sensilla of Tropiduchidae. **a** *Tropiduchus arisba*, Sa-PEPL, in lateral view. **b**–**h** *Numicia hulstaerti*. **b** Sub-apical location of the Sa-PEPL, length of the CH2. **c** SF-D with sensilla BSN1, BSN2 and PGSU2, SF-V with the BSN2 (sensillum no. 15). **d** Detailed view on *right side* (RS) of the dorsal sensory field: PGSM (sensillum no. 1), BSN2 (sensilla no. 2, 3, 13), PGSU2 (sensilla no. 4–12) and BSN1(sensillum no. 14). **e** Distribution of the PGSU2 in the dorsal sensory field, p pore. **f** Length of the PGSU2. **g**, **h** Length of the BSN2 and BSN1, a molting pore (ml) is visible

**Fig. 15:**
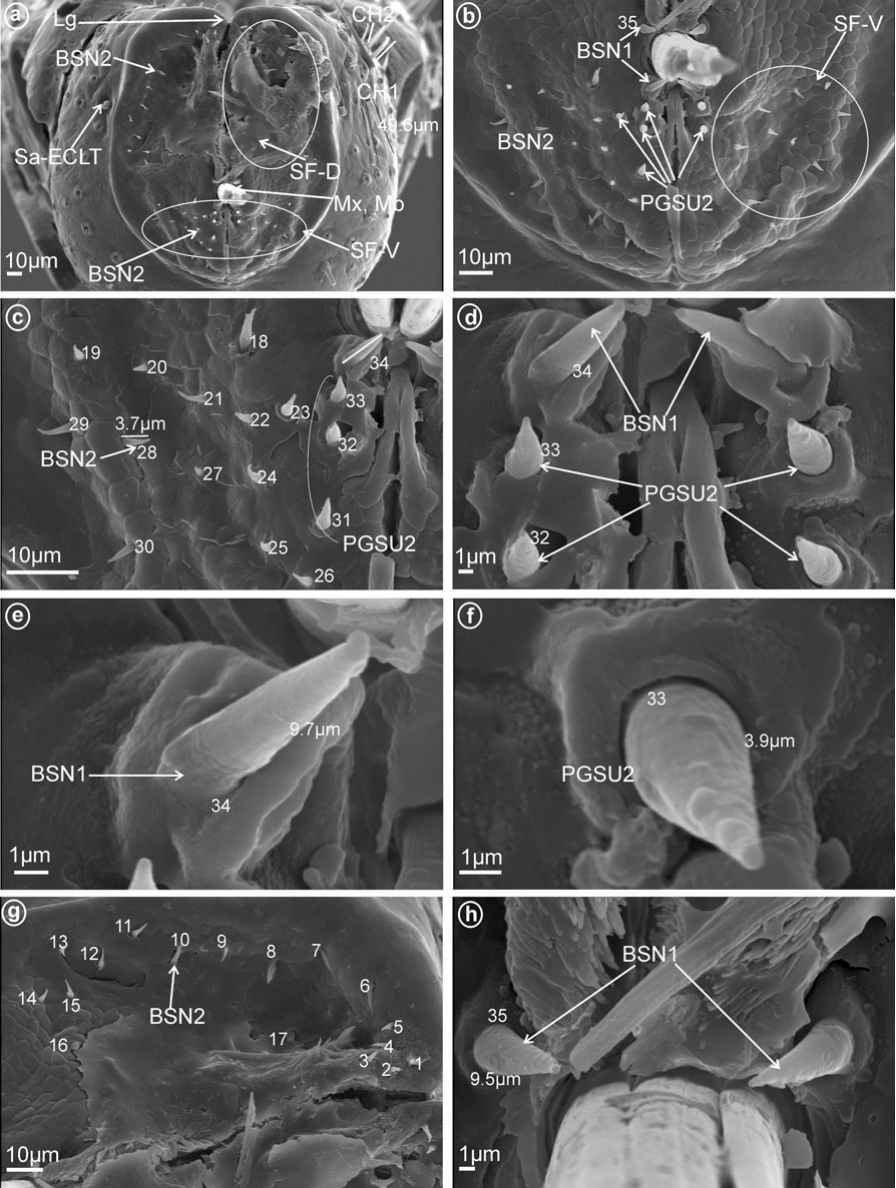
Types and arrangement of the labial sensilla of *Lophops africana* (Lophopidae). **a** Length of the CH1, CH2 and location of the Sa-ECLT, Lg, SF-D, SF-V, Mx, Md. **b** Ventral sensory field with visible BSN1, BSN2 and PGSU2. **c, d** Distribution of the sensilla in the ventral sensory field, BSN2 (sensilla no. 18–30), BSN1 (sensilla no. 34) and PGSU2 (sensilla no. 31–33). **e** Length of the BSN1 (sensillum no. 34). **f** Length of the PGSU2 (sensillum no. 33). **g** Numbers and location of BSN2 in the dorsal sensory field (sensilla no. 1–16). **h** BSN1 (sensillum no. 35) localized above the maxilla and mandibles

**Fig. 16:**
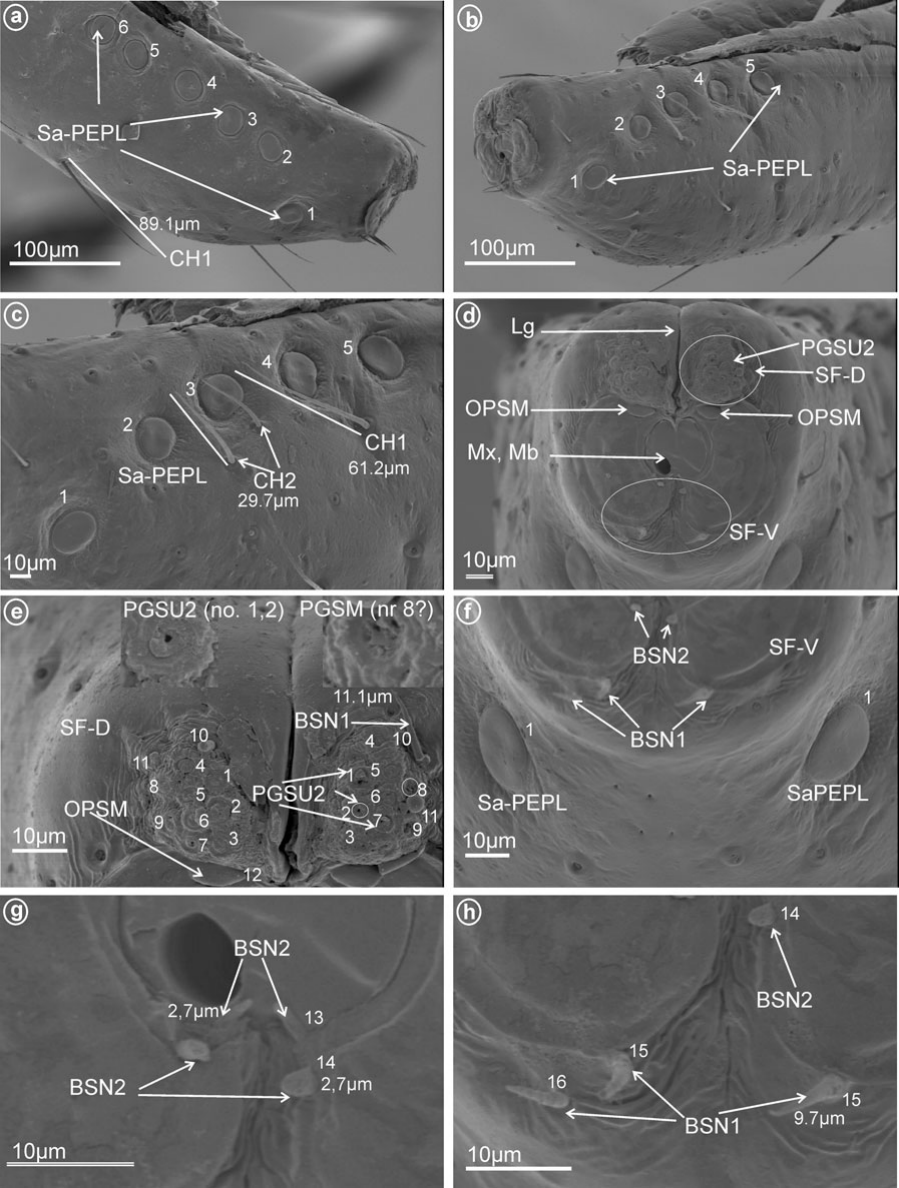
Types and arrangement of the labial sensilla of *Nogodina reticulata* (Nogodinidae). **a** Sa-PFPL (sensilla no. 1–6 on the *right side*). **b** Sa-PFPL (sensilla no. 1–5 on the *left side*). **c** Location of the Sa-PFPL and length of the CH2 and CH1. **d** Front view of the labial tip: PGSU2, Lg, SF-D, SF-V, Mx, Md, OPSM placed slightly below of the dorsal sensory field. **e** Dorsal sensory field with BSN1 (sensilla no. 10, 11), PGSM (sensillum no. 8 probably, there are five apertures at the base of this sensillum), PGSU2 (sensilla no. 1–7, 9, one aperture at the base in the case of these sensilla) and OPSM placed below the sensory field (sensillum no. 12). **f**–**h** Distribution of the sensilla in the ventral sensory field, BSN2 (sensilla no. 13, 14) and BSN1 (sensilla no. 15, 16)

**Fig. 17:**
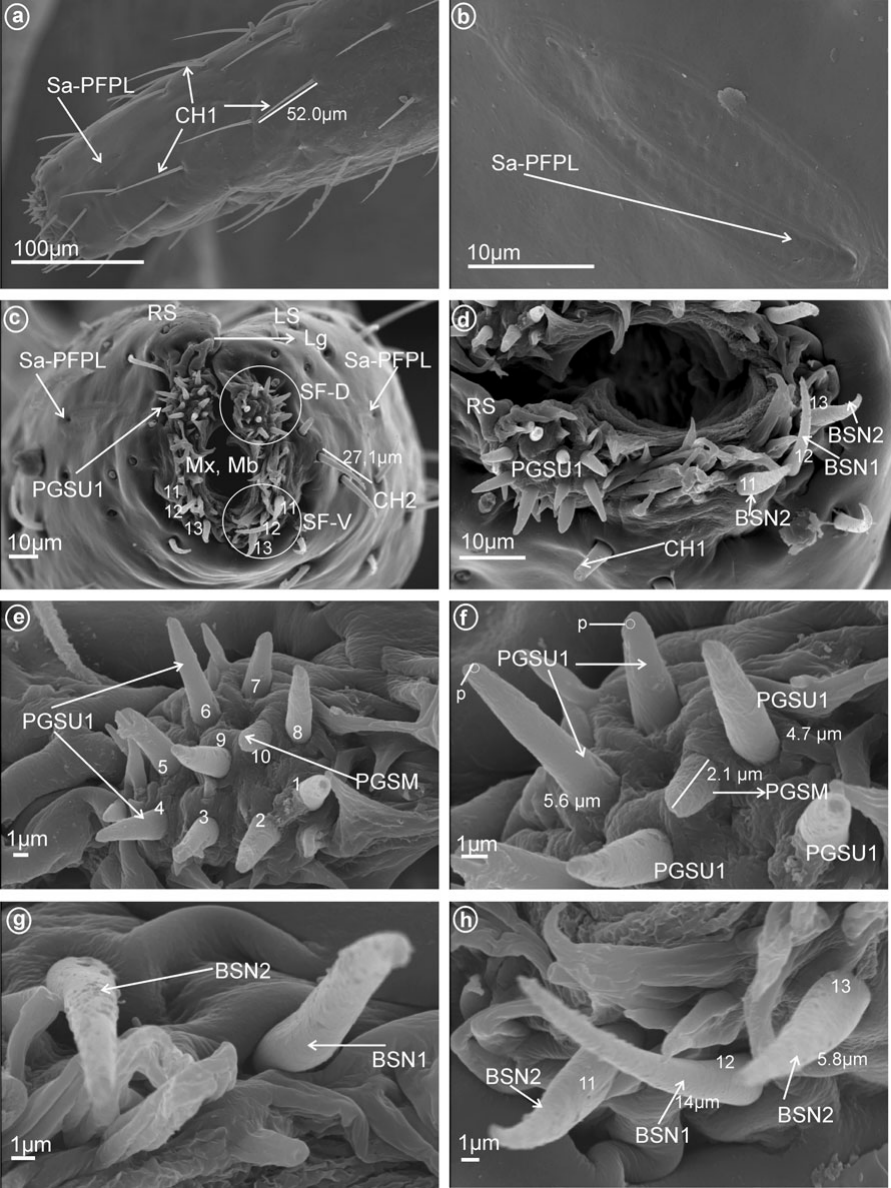
Types and arrangement of the labial sensilla of *Afronersia dionacea* (Dictyopharidae). **a** Length of the CH1, location of the Sa-PFPL. **b** Detailed view, Sa-PFPL sensillum, placoid flattened, multiporous. **c** Front view on the labial tip: Lg, SF-D, SF-V, Mx, Md, *right side* (RS) and *left side* (LS) of the tip, length of the CH2. **d** The dorsal and ventral sensory field with PGSU1 sensilla, BSN1 and BSN2 (sensilla no. 11, 13). **e, f** Distribution and length of the sensilla in the dorsal sensory field, PGSU1 (sensilla no. 1–9), PGSM (sensillum no. 10), p pore. **g**, **h** Distribution of the sensilla in the ventral sensory field BSN1 (sensillum no. 12) and BSN2 (sensilla no. 11, 13)
- *Short* (*20–30* μm) *and large* (*30–40* μm) *bristle-like sensilla, nonporous* (BRSN1, Fig. 1e–f). These sensilla were only found in Fulgoridae (Fig. 18c–g, sensilla no. 11–35).

**Fig. 18:**
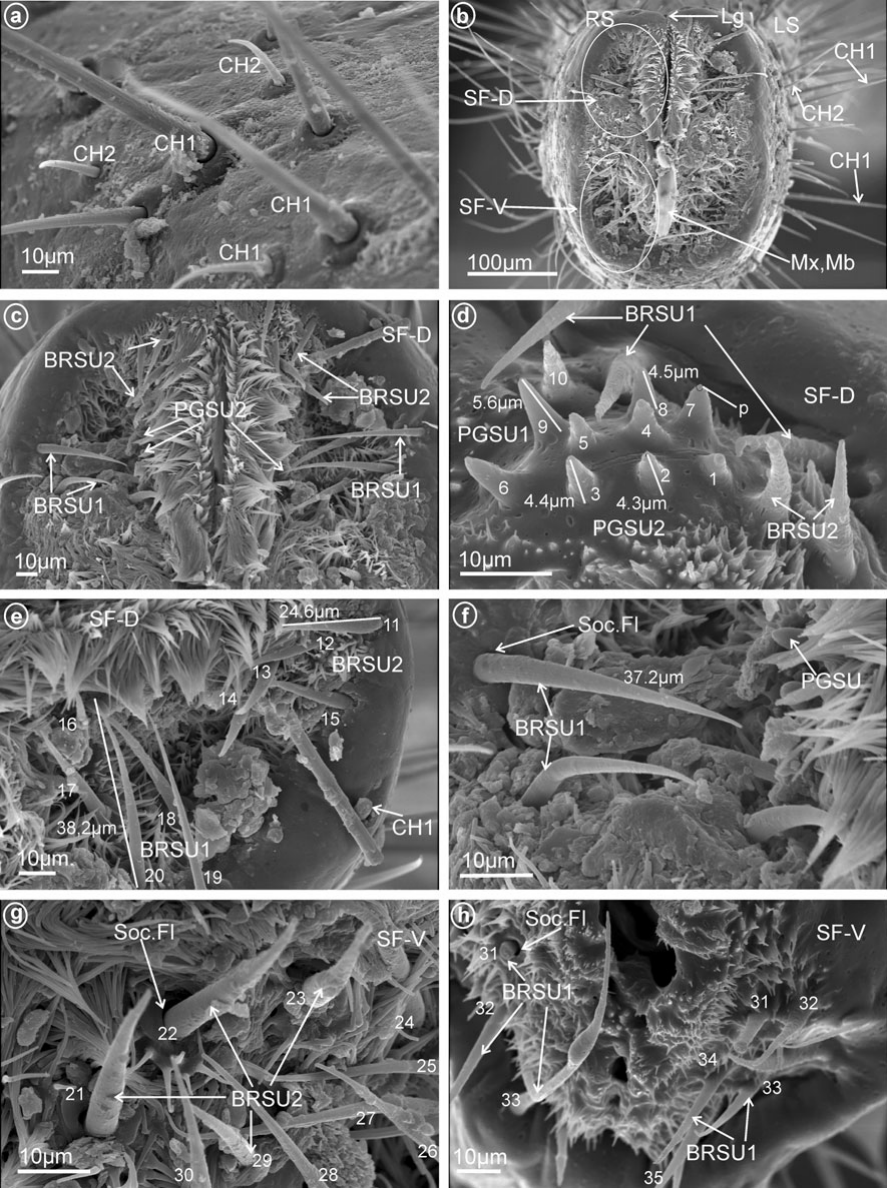
Types and arrangement of the labial sensilla of *Calyptoproctus* sp. (Fulgoridae). **a** Length of the CH1, CH2. **b** Front view of the labial tip: Lg, SF-D, SF-V, Mx, Md, RS *right side*, LS *left side* of the tip. **c** The dorsal sensory field with BRSU1 and BRSU2. **d** The dorsal sensory field with PGSU2 (sensilla no. 1–6), PGSU1 (sensilla no. 7–10). **e** Distribution and size of the sensilla on the dorsal sensory field, BRSU2 (sensilla no. 11–16) and BRSU1 (sensilla no. 17–20). **f** Length of the BRSU1. **g, h** Distribution of the sensilla in the ventral sensory field: BRSU2 (sensilla no. 21–24) and BRSU1 (sensilla no. 25–35), Soc.Fl flexible socket

#### Chemoreceptive MP sensilla: olfactory sensilla at the tip of labium

Olfactory sensilla are of various shapes and sizes and pierced by numerous pores. According to Altner and Prillinger (1980), these are wall-pore sensilla or multiporous sensilla as described by Zacharuk (1980). Five types of sensilla showing morphological characteristics connected with olfaction or olfacto-thermo reception are as follows:

- *Oval plate sensilla, multiporous* (OPSM, Fig. 1g). A pair of this type of sensillum were found only in Nogodinidae below the dorsal sensory field (Fig. 16d, e, sensillum no. 12).
- *Peg sensilla, multiporous* (PGSM, Fig. 2a). Cone-shaped with round tip sensillum with length ranging between 2.0 and 4.5 μm according to family. One pair of this type of sensillum was observed in Ricaniidae (Fig. 11f–h, sensillum no. 8, *L* = 3.2 μm), Flatidae (Fig. 13c, h, *L* = 2.5 μm), Tropiduchidae (Fig. 14d, sensillum no. 1), Nogodinidae (Fig. 16e, sensillum no. 8?—probably), Dictyopharidae (Fig. 17e, f, sensillum no. 10, *L* = 2.1 μm) and Fulgoridae (Fig. 18d, sensillum no. 8, *L* = 4.5 μm).
- *Peg sensilla, multiporous, complex* (PGSMC, Fig. 2b). This sensillum is divided into two parts by a furrow. Its length is 10.8 μm (Fig. 12h), and its tip is strongly pointed (Fig. 12f, h). Only one pair of this type of sensillum was observed, in Issidae (Fig. 12c, sensillum no. 8).
- *Cupola-shaped sensilla, multiporous* (CUSM, Fig. 2c). In this sensillum, the upper part is strongly enlarged relative to its base. The length of this sensillum is 1.5 μm (Fig. 8h). One pair of this kind of sensillum appeared to be specific for Meenoplidae (Fig. 8c, e–h, sensillum no. 8).
- *Dome-shaped sensilla, multiporous* (DSSM, Fig. 2d). Short sensilla (above 0.82 μm) with tip strongly flattened distinctly multiporius. One pair of this type of sensillum was situated on the dorsal sensory field among peg sensilla in Delphacidae (Fig. 5c, e, f, sensillum no. 8), Cixiidae (Fig. 6c, d, sensillum no. 8), Achilidae (Fig. 7e, f, sensillum no. 8) and Kinnaridae (Fig. 9d, f, h, sensillum no. 8).

#### Chemoreceptive UP sensilla: gustatory sensilla at the tip of the labium

These gustatory sensilla are bristles, hairs, pegs or just elevations of the cuticle, or a single flat cuticular area, all with a single terminal pore (TP-sensilla, uniporous) (Altner and Prillinger 1980). Peg sensilla have their side walls usually smooth but some have longitudinal ridges. They are divided into four types based on their length and shape of the tip as follows.

##### *Pit peg sensilla, uniporous* (PPSU, Fig. 2e **=** sensilla coeloconica)

These are typical peg-in-pit sensilla; they do not have any articulation and are characterized externally by a round aperture. The peg is oriented toward the inside of the pit in such a way that it is positioned in an exactly perpendicular position with respect to the external opening of the pit. One pair of these sensilla was observed in representatives of Delphacidae (Fig. 5e, f, sensillum no. 11), Cixiidae (Fig. 6d, sensillum no. 11), Achilidae (Fig. 7e, f, h, sensillum no. 10, *L* = 1.5 μm), Kinnaridae (Fig. 9d, f, h, sensillum no. 11) and Derbidae (Fig. 10b, e, f, sensillum no. 11).

##### *Peg sensilla, uniporous* (PGSU1, Fig. 2f)

**Fig. 19:**
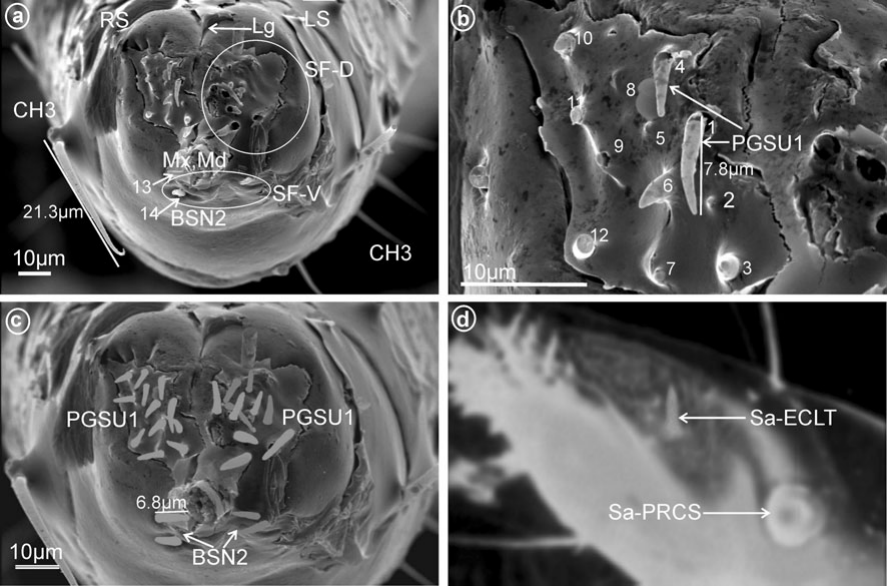
Types and distribution of the labial sensilla of Tettigometridae. *Tettigometra sulphurea*, **a** Length of the CH3 and location of the BSN2 (sensilla no. 13, 14), *right side* (RS) and *left side* (SL) of the tip, Lg, SF-D, SF-V, Mx, Md. **b** Dorsal sensory field with PGSU1 (sensilla no. 1–12). **c** Distribution of the PGSU1 and BSN2 sensilla on labial tip. **d** *Euphyonarthex phyllostoma*, Sa-ECLT elevated cone-like sensillum and Sa-PRCS placoid, rounded and convex sensillum

These cone-shaped sensilla are long (5.0–7.5 μm) with a rounded tip. This kind of the sensillum was found in Delphacidae (Fig. 5e, f, sensilla no. 2 and 9, *L* = 5.1 μm), Achilidae (Fig. 7e–h, sensilla no. 1 and 6, *L* = 7.25 μm, *L* = 5.25 μm), Ricaniidae (Fig. 11e–h, sensilla no. 1, 2, 7, *L* = 5.5 μm, *L* = 7.1 μm, *L* = 6.2 μm), Dictyopharidae (Fig. 17d–f, sensilla no. 1–9, *L* = 4.4 μm, *L* = 5.6 μm), Fulgoridae (Fig. 18c, d, sensilla no. 7–10, *L* = 5.6 μm) and Tettigometridae (Fig. 19b, c, sensilla no. 1–12, *L* = 7.8 μm).

##### *Peg sensilla, uniporous* (PGSU2, Fig. 2g)

Short cone-shaped sensilla (1.0–5.0 μm) with a rounded tip. This type of sensillum is common in many taxa: Delphacidae (Fig. 5e, f, g, sensilla no. 1–5, 7, *L* = 2.4 μm), Cixiidae (Fig. 6c–f, sensilla no. 1–7, 9, 10, *L* = 2.1 μm, *L* = 2.3 μm), Achilidae (Fig. 7e–h, sensilla no. 2, 3, 4, 5, 7, 9, *L* = 2.75 μm, *L* = 4.25 μm), Meenoplidae (Fig. 8e–h, sensilla no. 1–7, 9–10, *L* = 3.1 μm), Kinnaridae (Fig. 9d, f, g, sensilla no. 1–7, 9–10, *L* = 1.6 μm), Derbidae (Fig. 10b–f, sensilla no. 1–10, *L* = 3.5 μm, *L* = 2.6 μm), Ricaniidae (Fig. 11e–h, sensilla no. 3–6, 9, 10, *L* = 4.2 μm, *L* = 4.5 μm, *L* = 4.05 μm, *L* = 3.1 μm *L* = 3.2 μm), Flatidae (Fig. 13g, *L* = 2.3 μm), Tropiduchidae (Fig. 14b–f, sensilla no. 4–12, *L* = 3.2 μm), Lophopidae (Fig. 15b, c, f, sensilla no. 31–33, *L* = 3.9 μm), Nogodinidae (Fig. 16d, e, sensilla no. 1–7, 9) and Fulgoridae (Fig. 18d, sensilla no. 1–6, *L* = 4.3 μm, *L* = 4.4 μm).

##### *Clavate sensilla, uniporous* (CLSU, Fig. 2h)

These sensilla are similar to peg sensilla except distally enlarged (Fig. 12c, e, sensillum no. 8). Length 2.0 μm long (Fig. 12g). Only observed in Issidae.

#### Mechanoreceptive NP sensilla: tactile sensilla of the labium

Sensilla chaetica (CH) are the most abundant types of sensilla found on the labium in Fulgoromorpha (Fig. 3a). On the apical segment, they occur in different lengths and are articulated by a connective membrane to the cuticule (Fig. 3b). They exhibit grooved surfaces without pore (Fig. 3a, c). Based on their length, shape and surface morphology, three types are differentiated as follows:

- *Large* (45–>100 μm) *sensilla chaetica* (CH1)—The sensilla are long, relatively straight, gradually tapering and slightly curved at the tip (Fig. 3d, e).
- *Medium length* (20–45 μm) *sensilla chaetica* (CH2)—Shorter than CH1, with a fine tip and a strong base and running parallel to the surface (Fig. 3a, d).
- *Short* (1–10 μm) *sensilla chaetica* (CH3)—Short with sharp ends (Fig. 3d).

#### Olfactory and thermoreceptive MP sensilla: subapical labial sensilla

On each side and near the tip of the labium, there is one (or more) pair of sensilla that are multiporous, suggesting an olfactory and thermo-reception function. According to their shape, three main types are recognized as follows:

- an *elevated, cone-like to tubular sensilla* (Sa-ECLT) of 15.5 μm length (Fig. 4a–c).
- a *branched or multilobated tubular sensilla* of 8.0 um length (Sa-TEBM) (Fig. 4d).
- a *placoid flattened sensilla* (Sa-PFPL), peg-like, slightly convex or concave, surrounded by a double furrow (Fig. 4e–i). In Flatidae, the surface of the placoid (Sa-PMPL) has numerous minute 1 μm lobes (Figs. 4j, 13a, b).

## Discussion

This paper presents the first comparative analysis of the labial sensilla in planthoppers. Although the studied examples of each family are small, some new and interesting general features are shown.

### The planthopper labial sensory ground plan

The sensory equipment in planthoppers consists of well-known mechanoreceptor sensilla chaetica plus specialized areas/structures: the paired subapical sensory labial organs and the apical sensory labial area. With respect to the latter, the total number of sensilla at the tip of the labium allows us to recognize two main groups in Fulgoroidea. The first group exhibits 12–19 sensilla pairs (12 in meenoplids, derbids and achilids, 13 in cixiids, delphacids, dictyopharids and kinnarids, 14 in tettigometrids, 15 in tropiduchids and issids, 16 in nogodinids and 19 in ricaniids), and the second group shows a greater number of pairs (32 in flatids and 35 in lophopids and fulgorids). See Table 2.

According to the currently accepted phylogenies of Fulgoroidea (reviewed in Bourgoin et al. 1997; Urban and Cryan 2007), it seems that the first group of families (noted above) is usually placed at the base of the phylogeny and therefore might represent the plesiomorphic condition. With the exception of the 6 sensilla basiconica (BSN1) observed in the ricaniid representative (that probably represent a specialized condition), the planthopper ground plan should therefore be represented by two pairs of sensory fields:

1. dorsal paired sensory fields with 11 pairs of sensilla (10 peg-like pairs + 1 specialized pair dome or cupola-like)
2. ventral impair or paired sensory field with 2 pairs of sensilla basiconica.

The second group of families (noted above) is therefore to be regarded as advanced. The Fulgoridae have retained the 10 dorsal peg-like pairs (as in dictyopharids) completed by a great number of bristle-like sensilla (10 dorsal and 15 ventral pairs). Two families have increased their number of sensilla basiconica, that is, Lophopidae, with only sensilla basiconica distributed in 35 pairs, a pattern approached by the flatids with 17 peg-like and 17 sensilla basiconica pairs.

It is probable that the eleventh specialized pair of dorsal sensilla (either clavate, dome-like or cupola-like) should be regarded as evolved from a more plesiomorphous peg-like sensillum.

This planthopper ground plan can be compared to what is already known in other Hemiptera groups. In Sterrnorhyncha, the labial tip in Aleyrodoidea has seven pairs of sensilla (chemosensitive and contact chemoreceptive) according to a scheme apparently similar among species and sexes (Walker and Gordh 1989); in Psylloidea, four pair peg-like sensilla have been reported, but their exact function remains unknown (Garzo et al. 2012), and in Aphidoidea, only mechanoreceptive sensilla (eight or seven pairs) are present (Wesler 1977; Tjallingii 1978).

In other Auchenorrhyncha, a trend toward a reduction of sensilla seems to have taken place (Leopold et al. 2003; Backus 1988), but a significant variation in type and distribution of sensilla has also been observed (Brożek in prep.).

In Heteroptera, the labial sensilla have been studied in more than 70 species (Beck et al. 1958; Schoonhoven and Henstra 1972; Peregrine 1972; Cobben 1978; Avé et al. 1978; Gaffal 1981; Backus 1988; Rani and Madhavendra 1995; Ventura et al. 2000; Ventura and Panizzi 2005; Brożek 2008; Brożek and Chłond 2010).

In most of these species, the sensilla are more or less numerous and of different shape and size, allowing us to recognize both chemosensitive sensilla (gustatory, contact-chemoreceptive) and mechanoreceptive sensilla. In Reduviidae, it has been shown that interspecific variability and intraspecific similarity in the shape and numbers of labial sensilla have potential usefulness as taxonomic/diagnostic characters (Català 1996; Brożek and Chłond 2010).

The above results confirm a ground plan of seven pairs of sensilla in Sternorrhycnha to a richer and more diverse sensory equipment in Auchenorrhycnha, particularly in planthoppers and Heteroptera as noted by Backus (1988).

### The labial sensilla in planthoppers

As noted above, this study reveals for the first time the diversity of sensory structures on the labial tip and their grouping into sensory fields in Fulgoromorpha. More taxa need to be studied before trying to present some general scheme at the family level, but some specific morphological types and their distribution seem characteristics of certain groups.

- *Disparity of the apical labial sensilla*: From the ground plan proposed for planthoppers (10 peg-like dorsal pairs + 1 dome or cupola-like dorsal pair + 2 sensilla basiconica ventral pairs), the great disparity reported in Table 2 suggests that it is the result of several independent evolutionary events from one plesiomorphic sensilla type, probably peg-like. Only more data/taxa analyzed in a precise phylogenetical framework will allow us to understand how theses types have evolved.
- *Distribution of the apical labial sensilla*: At this stage, and according to their distribution, three patterns can be suggested.

#### The general cixiid pattern (Cixiidae, Delphacidae, Achilidae, Meenoplidae, Kinnaridae, Dictyopharidae, Derbidae and Tettigometridae)

Apical sensilla are clearly arranged in two sensory fields (Table 3). The number of the sensilla ranges from 12 to 13 pairs. According to the samples, only minor differences are observed and concern the type and number of sensilla. Among the more specific types of sensilla, the dome-shaped multiporous sensilla are absent in Meenoplidae and probably replaced by its homologous cupola-shaped sensilla. The peg-in-pit uniporous sensillum present in Delphacidae, Cixiidae, Achilidae and Kinnaridae has not been seen in Meenoplidae. In Dictyopharidae and Tettigometridae, the uniporous peg sensilla (PGSU2) are replaced by longer sensilla (PGSU1). Sensilla basiconica (BSN1, BSN2) are found in the above families only in the ventral sensory field.

**Table 3:** Distribution of the different apical sensilla in selected fulgoromorphan families

Families and species	Types of the sensilla and their distribution	Scheme of the apical tip of labium
Delphacidae *Peregrinus maidis* (Ashmead, 1890) See Fig. 5a–f	Dorsal sensory field (SF-D) convex: 8 PGSU2, uniporous peg sensilla, short 1 PGSU1, uniporous peg sensilla, long 1 PPSU, uniporous peg-in-pit sensilla 1 DSSM, multiporous dome-shaped sensilla Ventral sensory field (SF-V)—unpair, convex: 2 BSN2, sensilla basiconica, short	
Cixiidae *Myndus taffini* Bonfils, 1983 Fig. 6a–d *Brixidia boukokensis* Synave, 1958 Fig. 6e–f	Dorsal sensory field (SF-D) concave: 9 PGSU2, uniporous peg sensilla, short 1 DSSM, multiporous dome-shaped sensilla 1 PPSU, uniporous peg-in-pit sensilla Ventral sensory field (SF-V) concave: 2 BSN2, sensilla basiconica, short	
Achilidae *Ballomarius kawandanus* Fennah 1950 Fig. 7c–f	Dorsal sensory field (SF-D) convex: 6 PGSU2, uniporous peg sensilla, short 2 PGSU1, uniporous peg sensilla, long 1 DSSM, multiporous dome-shaped sensilla 1 PPSU, uniporous peg-in-pit sensilla Ventral sensory field (SF-V) convex: 1 BSN1, sensilla basiconica, long 1 BSN2, sensilla basiconica, short	
Meenoplidae *Kermesia immaculata Muir, 1927, Fig. 8a *Nisia nervosa* (Motchulsky, 1863) Fig. 8b–h	Dorsal sensory field (SF-D) convex: 9 PGSU2, uniporous peg sensilla, short 1 CUSM, multiporous cupola-shaped sensilla Ventral sensory field (SF-V) unpair, convex: 2 BSN2, sensilla basiconica, short	
Kinnaridae *Southia capnorhina Fennah, 1980, Fig. 9a *Atopocixius major Fennah, 1945, Fig. 9b *Nesomircixia insularis* Synave, 1958 Fig. 9c–h	Dorsal sensory field (SF-D) convex: 9 PGSU2, uniporous peg sensilla, short 1 DSSM, multiporous dome-shaped sensilla 1 PPSU, peg-in-pit uniporous sensillum Ventral sensory field (SF-V) convex: 1 BSN1, sensilla basiconica, long 1 BSN2, sensilla basiconica, short	
Derbidae *Proutista fritillaris* (Boheman,1838) Fig. 10b–g	Dorsal sensory field (SF-D) is divided into flat fields A and B: 4 PGSU2, uniporous peg sensilla, short (SF-D-A) 6 PGSU2, uniporous peg sensilla, short (SF-D-B) 1 PPSU, peg-in-pit uniporous sensillum Ventral sensory field (SF-V) unpair, flat: 1 BSN2, sensilla basiconica, short	
Ricaniidae *Pochazia fuscata* Fabricius, 1803 Fig. 11a–h	Dorsal sensory field (SF-D) concave: 6 PGSU2, uniporous peg sensilla, short 3 PGSU1, uniporous peg sensilla, long 1 PGSM, multiporous peg sensilla 6 BSN1, sensilla basiconica, long Ventral sensory field (SF-V) concave: 1 BSN1, sensilla basiconica, long 2 BSN2, sensilla basiconica, short	
Issidae *Trienopa paradoxa* (Gerstaecker, 1892) Fig. 12b–c	Dorsal sensory field (SF-D) convex: 1 PGSMC, complex, multiporous peg sensilla, 8 CLSU), uniporous clavate sensilla 4 BSN2, sensilla basiconica, short Ventral sensory field (SF-V) convex: 2 BSN1, sensilla basiconica, long	
Flatidae *Flatia* sp. (Schmidt, 1912) Fig. 13c–h	Apical sensilla are scattered widely on the labial tip. The boundary between the upper and ventral sensory fields is almost not visible because the sensilla occupy the entire tip surface of the labium. Dorsal sensory field (SF-D) concave: 16 PGSU2, uniporous peg sensilla, short 1 PGSM, multiporous peg sensillum Ventral sensory field (SF-V) concave: 3 BSN2, sensilla basiconica, short 12 BSN1, sensilla basiconica, long are distributed on the edge, around the dorsal sensory field	
Tropiduchidae *Tropiduchus arisba Fennah, 1958 Fig. 14a *Numicia hulstaerti* (Synave, 1962) Fig. 14b–d *Tambinia sp. Stål, 1859 *Kalitaxilana sp. Kirkaldy, 1901	Dorsal sensory field (SF-D) concave is more extended and reaches laterally to the mandibular (Md) and maxillary stylets (Mx): 9 PGSU2, uniporous peg sensilla, short 1 PGSM, multiporous peg sensila 3 BSN2, sensilla basiconica, short 1 BSN1, sensilla basiconica, long Ventral sensory field (SF-V) concave: 1 BSN2, sensilla basiconica	
Lophopidae *Lophops africana* (Schmidt, 1912) Fig. 15a–h	Dorsal sensory field (SF-D) concave: 17 BSN2, sensilla basiconica, short 1 BSN1, sensilla basiconica, long between the dorsal sensory field and the maxillary and mandibular stylets Ventral sensory field (SF-V) concave: 3 PGSU2, uniporous peg sensilla, short 13 BSN2, sensilla basiconica, short 1 BSN1, sensilla basiconica, long	
Nogodinidae *Nogodina reticulata* Fabricius, 1803 Fig. 16d–h	Dorsal sensory field (SF-D) convex: 8 PGSU2, uniporous peg sensilla, short 1 PGSM, multiporous peg sensilla 2 BSN1, sensilla basiconica, long 1 OPSM, multiporous oval plate sensilla, they are between the dorsal sensory field and the maxillary and mandibulary stylets Ventral sensory field (SF-V) convex: 2 BSN1, sensilla basiconica, long 2 BSN2, sensilla basiconica, short	
Dictyopharidae *Afronersia dionacea* Fennah, 1958 Fig. 17c–h	Dorsal sensory field (SF-D) convex: 9 PGSU1, uniporous peg sensilla, long 1 PGSM, multiporous peg sensillum Ventral sensory field (SF-V) convex: 2 BSN2, sensilla basiconica, short 1 BSN1, sensilla basiconica, long	
Fulgoridae *Calyptoproctus* sp. Fig. 18b–h	Sensilla are numerous and densely arranged in both sensory fields. Dorsal sensory field (SF-D) convex: 6 PGSU2, uniporous peg sensilla, short 3 PGSU1, uniporous peg sensilla, long 1 PGSM, multiporous peg sensilla 6 BRSN2, uniporous, bristle-like sensilla, short 4 BRSN1, uniporous, bristle-like sensilla, long Ventral sensory field (SF-V) convex: 4 BRSN2, uniporous, bristle-like sensilla, short 11 BRSN1 uniporous, bristle-like sensilla, long	
Tettigometridae *Tettigometra sulphurea* Mulsant & Rey, 1855 Fig. 19a–c *Euphyonarthex phyllostoma Schmidt, 1912 Fig. 19d	Dorsal sensory field (SF-D) convex: 12 PGSU1, uniporous, peg sensilla, long Ventral sensory field (SF-V) convex: 2 BSN2, sensilla basiconica, short	

On each sensory field (right and left), the sensilla are arranged symmetrically with the number of sensilla given for one side

In Derbidae, apical sensilla are distinctly grouped in three areas, a very probable apomorphic condition. Twelve pairs of sensilla are observable. The dorsal sensory field is subdivided into two fields (marked as A and B, Fig. 10b). The lower one (B) is located laterally in relation to the maxillae and mandibles. A characteristic feature is the presence of the uniporous peg-in-pit sensillum (PPSU) in the lower dorsal field. Sensilla basiconica (BSN2) occur only in the ventral sensory field. This specialized derbid pattern has very probably evolved from the general cixiid one.

#### The general issid pattern (Issidae, Nogodinidae, Tropiduchidae, Ricaniidae and Lophopidae?)

Apical sensilla are distinctly grouped into two sensory fields. A number of the sensilla range from 15 to 23 pairs. Sensilla basiconica (BSN1, BSN2) are present in the dorsal and ventral sensory fields. In the Tropiduchidae, Nogodinidae and Ricaniidae representatives, a multiporous peg sensillum (PGSM) is observed, probably homologous with the issid multiporous complex peg sensillum (PGSMC). In addition, in the Issidae species, a specific shaped uniporous peg sensilla (clavate with enlarged tip) is found.

A unique arrangement of two sensilla located between the dorsal sensory field and the opening of the maxillae and mandibles is observed in the Nogodinidae (OPSM). A similar position of the BSN1 is found in Lophopidae which is also unique in its uniporous peg sensilla in the ventral sensory field and the great number of sensilla basiconica in the ventral sensory field. This represents a specialized pattern (lophopid pattern) that has probably evolved from the issid one.

#### The specialized flatid and fulgorid patterns (Flatidae, Fulgoridae)

In these families, apical sensilla are arranged in two sensory fields, but the boundaries between the fields are difficult to trace due to the numerous sensilla (32–35 pairs) that cover the entire labial tip surface, especially sensilla basiconica and bristle-like sensilla. Further studies are necessary to evaluate these patterns that have probably evolved independently.

#### Sensilla chaetica

Recognition of 3 types of sensilla chaetica according to the length is an unexpected result. The medium type (CH2) seems the more largely distributed within the samples and might represent the plesiomorphic type from which evolutionary specialization has occurred toward shorter (CH3) or longer sensilla (CH1). In other parts of the insect body, mechanosensitive sensilla are of wide occurrence, so it is possible that the subdivisions proposed here may need to be revised when these are studied further.

In the studied sample, the distribution of the three types of sensilla on the labial tip differs greatly as follows. Only type CH3 was present in Meenoplidae (Fig. 8b) and only type CH2 occured in Achilidae (Fig. 7a), Kinnaridae (Fig. 9c), Tropiduchidae (Fig. 14b) and Tettigometridae (Fig. 19a), while both these types were present in Delphacidae (Fig. 5a) and Cixiidae (Fig. 6a). Types CH1 and CH2 were noticed in Ricaniidae (Fig. 11b), Issidae (Fig. 12a), Lophopidae (Fig. 15a), Nogodinidae (Fig. 16c), Dictyopharidae (Fig. 17a, c) and Fulgoridae (Fig. 3a, d, Fig.18a, b). All three types were observed in Derbidae (Fig. 10a) and Flatidae (Fig. 13a).

### The subapical labial sensory organ

Sogawa (1977) was the first to report the presence of a paired subapical sensory organ (wrongly named ‘labial palpi’) along the sides of the labium in some delphacids, represented by a pair of multiporous branched sensilla sunken into a small pit. This subapical sensory organ has since been found in most Fulgoromorpha families by Cobben (1988), Liang (2005) and in this study and also found in the 1st larval stage in Cixiidae (Thierry Bourgoin, unpublished data).

All types observed are multiporous sensilla. The elevated, cone-like or tubular sensilla types were found in Cixiidae (Figs. 4a, 6a, b, e), Meenoplidae (Figs. 8a, b, 4b), Kinnaridae (Fig. 9a, b), Lophopidae (Figs. 4c, 15a) or Tettigometridae (Fig. 19d). The tubular branched shape (Fig. 5b) could represent an autapomorphic character for Delphacinae (Cobben, 1988). The last placoid flattened type was observed in Achilidae (Figs. 4e, 7a, b), Dictyopharidae (Figs. 4f, 17a, b), Tropiduchidae (Figs. 4g, 14a, b), Ricaniidae (Figs. 4h, 11b) and Nogodinidae (Fig. 4i, 16a, b). In Flatidae, the surface of the placoid was not flattened but multilobated with numerous minute lobes (Figs. 4j, 13a, b). Intriguingly, in several representatives, the sensilla were present in more than one pair, for example, 5 of them (placoid type) were found in Nogodinidae (Fig. 16a, b). In Tettigometridae (*E. phyllostoma*), 2 pairs of sensilla were observed, the anterior one being cone-like (Sa-ECLT) and the posterior represent rounded placoid convex sensilla (Sa-PRCS) (Fig. 19d).

In other Hemiptera, the subapical labial sensory organ seems to be absent in the Cicadomorpha (Cobben 1988), but a very similar cixiid-like structure, called the “baton-shaped structure”, has been reported on the lateral subapical part of the labium of the bed-bug (*Cimex hemipterus*) by Singh et al. (1996, Fig. 3e). In the first conservative and parsimonious approach to these observations, we consider that the paired subapical organs are homologous in all planthoppers taxa. This same organ is rather polymorphic and can evolve into quite different shapes or might disappear. Again, according to the currently accepted phylogeny of the group (reviewed in Bourgoin et al. 1997; Urban and Cryan 2007), the organ comprising cone-shaped or tubular sensilla would therefore represent the plesiomorphic state (as in *Cimex hemiptera*), from which at least two new apomorphic-shaped sensilla have evolved: the multi-branched Delphacinae one and the placoid one, each obviously arising independently in several planthopper lineages. The apparent absence of these sensory structures in Derbidae and Fulgoridae needs to be confirmed. The presence of the organ cannot be considered as a possible synapomorphy for the clade [Cixiidae + Delphacidae + Achilixiidae + Achilidae] as stated by Liang (2005) but rather a symplesiomorphy for all Fulgoromorpha, if not a synapomorphy for all the Neohemiptera clade (Fulgoromorpha + Heteropterodea) (Sorensen et al. 1995). However, more morphological studies of the Hemiptera labium in all these groups are needed before positive conclusions can be reached. As suggested already by Cobben (1988), it is possible that, at least for the trunk-feeding fulgorids, the absence of the subapical sensory organs (if confirmed) could be linked to host-plant preference.

### Labial sensilla and their function in planthoppers

#### The labium and the olfactory function

It is generally accepted that odorant substances, including sex pheromones and host-plant volatiles, diffuse through the wall pores of the multiporous sensilla into the sensillar lymph and are transferred to receptors on the dendrites of specialized neurons by special binding proteins (Leal 2005). In most insects, these olfactory multiporous sensilla occur mainly on the antennae and often on the palps, when these are present. In planthoppers, the characteristic sensory plate organs of the pedicel antenna (Bourgoin and Deiss 1994, reviewed in Stroiński et al. 2011), are very probably in charge of the olfactory function.

In the current study, five multiporous sensilla without flexible sockets have been detected on the labium: the dome-shaped sensilla, the cupola-shaped sensilla, the oval plate sensilla, the peg and the complex peg sensilla. Similar sensilla are known in other hemipteran taxa such as Aleyrodidae (Walker and Gordh 1989) or Heteroptera (Schoonhoven and Henstra 1972; Peregrine 1972; Gaffal 1981), but sometimes also reported as absent (Avé et al. 1978; Hatfield and Frazier 1980; Rani and Madhavendra 1995; Brożek and Chłond 2010). As such types of sensilla are reputed to have an olfactory function (Slifer 1970; Zacharuk 1980; Steinbrecht 1984; Hallberg et al. 2003; Kristoffersen et al. 2006; Onagbola and Fadamiro 2008), one can say that the Hemiptera labium assists the antenna in this function, but the association has not yet been fully studied.

#### The labium and the gustatory function

In planthoppers, the dominant group of the sensilla at the tip of the labium is uniporous sensilla: peg sensilla (PGSU1, PGSU2) and, less commonly, peg-in-pit (PPSU) and clavate sensilla (CLSU). The different shapes of the uniporous sensilla on the labium are probably linked to the detection of both physical and various chemical stimuli that are non-volatile or have low volatility, and also through close or direct host contact. All sensilla are located in the dorsal sensory field and belong to the group of gustatory sensilla.

After reviewing Chapman (1982), Backus (1988) pointed out that in heteropteran bugs, information about the volatiles emanating from the surface of plants, and their interaction with the insect cuticle, occurs during the exploration of the plant surface through antennation with the antennal flagellum. It suggests that this occurs through the contact-chemoreceptive sensilla with a gustatory function. In contrast, planthoppers do not antennate the plant during surface exploration, their antennae being too small to reach the plant surface. Accordingly, it is likely that the function of plant surface exploration is transferred to the apex of the labium in these insects, during dabbing the plant surface (Backus 1988; Ventura and Panizzi 2005). This evolution belongs to a form of exaptation (an additional new function for a plesiomorphic structure). How this evolution took place remains to be studied but obviously will need to be studied conjointly with the characteristic sensory plate organs of planthopper antennae.

#### The labium and the contact function

Ultrastructural studies show that sometimes a mechanoreceptive dendrite may be associated with sensilla that function as contact-chemoreceptive sensilla (Zacharuk 1980; Foster et al. 1983b). Such sensilla, with terminal pore and flexible socket, were not observed during our study, while Foster et al. (1983b) and Backus (1985) mentioned that some of sensilla should be regarded as mechano-chemoreceptive. However, there are difficulties in identifying the two types of sensilla which relies on the presence or absence of the terminal pore. Currently, it is uncertain whether the sensilla basiconica and bristle-like sensilla (BSN1, BSN2, BRSN1, BRSN2) have a terminal pore, although all are embedded in a flexible socket.

Morphological evidence on the labial tip of other hemipterans suggested that contact-chemoreceptive sensilla occur rather frequently in Heteroptera (Schoonhoven and Henstra 1972; Avé et al. 1978; Hatfield and Frazier 1980; Gaffal 1981; Rani and Madhavendra 1995; Rani 2009; Baker et al. 2008) and Aleyrodidae (Walker and Gordh 1989) and Psyllidae (Garzo et al. 2012). Conversely, sensilla basiconica (BSN1, BSN2) that are the second most abundant type of sensillum found on the tip of the labium in Fulgoromorpha are typical mechanoreceptive sensilla. They are generally located in the ventral sensory field below the maxillar and mandibular stylets. They probably assist in positioning the labium during feeding.

#### The subapical labial sensory organ and its function

In the delphacids *N. lugens* and *P. maidis*, Foster et al. (1983b) and Backus (1985) reported the presence of many pores on the sensilla of the subapical labial organ, but with a slightly different structure and distribution of dendrites in comparison with the multiporous sensillum on the tip of labium. Their function has not yet been identified.

In several different insects, it has been suggested that sensilla that are recessed from the antennal surface and located within cavities can be involved in measuring humidity and temperature (Steinbrecht 1984; Stange and Stowe 1999; Onagbola and Fadamiro 2008) and may play a role in preventing desiccation (Kristoffersen et al. 2006). Moreover, similar morphological structures (multilobed or branched sensilla) have also been suggested as possible hydro-receptive or olfacto-receptors (K3 type sensillum) in Coleoptera antennae (Roth and Willis 1951; Meinecke 1975). Accordingly, we provisionally regard them as complex dual functioning sensory organs. However, the placoid type observed in several taxa might operate only for more specialized olfactory functions, as on the antennal flagellum of cicadas (Klein et al. 1988).

## Conclusions

The diverse type, number and distribution of labial sensilla appear much more important than previously supposed. This new set of characters, studied here, allows for the following conclusions:

1. According to the morphological characteristics of the labium sensilla, it appears that they can provide planthoppers with information about tactile, olfactive and gustative stimuli when near or in contact with the plant. The gustatory function appears to be more recently evolved in Auchenorrhyncha compared to other Hemiptera that explore their host-plants by tapping with their antennae. Obviously, these findings will have to be put in perspective with the feeding behavior and diversity of trophic patterns in planthoppers and their host-plant as already observed (Attié et al. 2008).
2. Clearly, this new set of characters brings some interesting evolutionary signal to further studies in taxonomy (identification) and phylogeny of the Fulgoromorpha. While conclusions at this stage are obviously premature, one can already see several issues for further studies:
  From a phylogenetical perspective, will the special types of sensilla observed be representative and autapomorphic of the groups in which they have been found? Is the presence of sensilla basiconica (BSN1, BSN2) in the dorsal sensory field a characteristic only of the higher fulgoroidea families? With more samples studied, will the two main patterns observed be broken into more specialized subdivisions and therefore what phylogenetical value will these smaller divisions have? At a higher level and with a bigger dataset what will be the implications of the presence/absence of the subapical labial sensory organ have for the hemiptera phylogeny?
  From a behavioral perspective: what is the functional signification of the separate pair of sensilla between the dorsal and the ventral sensory field of the tip of the labium as observed in the nogodinid and lophopid representatives and of the three sensory fields in derbids? Will they have any value in constructing the phylogeny of these groups? Several representatives show a trend to the multiplication of the sensory units at the tip of the labium, is there any link between this morphology and diet? Can the absence of the subapical sensilla in Fulgoridae really be linked to a different mode of feeding (tree feeding) in these insects?
